# Immunogenicity and protection efficacy of a COVID-19 DNA vaccine encoding spike protein with D614G mutation and optimization of large-scale DNA vaccine production

**DOI:** 10.1038/s41598-024-64690-5

**Published:** 2024-06-15

**Authors:** Aytül Gül, Sedef Erkunt Alak, Hüseyin Can, Muhammet Karakavuk, Gülay Korukluoğlu, Ayşe Başak Altaş, Ceren Gül, Tuğba Karakavuk, Ahmet Efe Köseoğlu, Hivda Ülbeği Polat, Hilal Yazıcı Malkoçoğlu, Arzu Taş Ekiz, İrem Abacı, Özge Aksoy, Hakan Enül, Cumhur Adıay, Serdar Uzar, Fahriye Saraç, Cemal Ün, Adnan Yüksel Gürüz, Ayşe Gülten Kantarcı, Hasan Akbaba, Gülşah Erel Akbaba, Habibe Yılmaz, Aysu Değirmenci Döşkaya, Meltem Taşbakan, Hüsnü Pullukçu, Ercüment Karasulu, Şaban Tekin, Mert Döşkaya

**Affiliations:** 1 Vaccine Development Application and Research Center, Ege University, 35100 İzmir, Türkiye; 2 Department of Bioengineering, Faculty of Engineering, Ege University, İzmir, Türkiye; 3 Department of Bioengineering, Graduate School of Natural and Applied Sciences, Ege University, İzmir, Türkiye; 4 Department of Biology, Molecular Biology Section, Faculty of Science, Ege University, İzmir, Türkiye; 5 Department of Vaccine Studies, Institute of Health Sciences, Ege University, İzmir, Türkiye; 6 Ödemiş Vocational School, Ege University, İzmir, Türkiye; 7 Republic of Türkiye, General Directorate of Public Health, Ministry of Health, National Virology Reference Central Laboratory, Ankara, Türkiye; 8 Department of Medical Microbiology, Ankara Bilkent City Hospital, University of Health Sciences, Ankara, Türkiye; 9 Department of Biotechnology, Graduate School of Natural and Applied Sciences, Ege University, İzmir, Türkiye; 10 Department of Environmental Microbiology and Biotechnology, Faculty of Chemistry, Duisburg-Essen University, Essen, Germany; 11 TÜBİTAK Marmara Research Center, Vice Presidency of Life Sciences, Kocaeli, Türkiye; 12 Pendik Veterinary Control Institute, İstanbul, Türkiye; 13 Department of Parasitology, Faculty of Medicine, Ege University, İzmir, Türkiye; 14 Department of Pharmaceutical Biotechnology, Faculty of Pharmacy, Ege University, İzmir, Türkiye; 15 Department of Pharmaceutical Biotechnology, Faculty of Pharmacy, İzmir Katip Çelebi University, İzmir, Türkiye; 16 Department of Pharmaceutical Biotechnology, Faculty of Pharmacy, Trakya University, Edirne, Türkiye; 17 Department of Infectious Diseases, Faculty of Medicine, Ege University, İzmir, Türkiye; 18 Ege University Research and Application Center of Drug Development and Pharmacokinetics, İzmir, Türkiye; 19 Department of Basic Medical Sciences, Medical Biology, Faculty of Medicine, University of Health Sciences, İstanbul, Türkiye

**Keywords:** SARS-CoV-2, DNA vaccine, K18-hACE2 transgenic mice, Large-scale production, Upstream, Downstream, Drug discovery, Microbiology

## Abstract

Severe acute respiratory syndrome coronavirus 2 had devastating consequences for human health. Despite the introduction of several vaccines, COVID-19 continues to pose a serious health risk due to emerging variants of concern. DNA vaccines gained importance during the pandemic due to their advantages such as induction of both arms of immune response, rapid development, stability, and safety profiles. Here, we report the immunogenicity and protective efficacy of a DNA vaccine encoding spike protein with D614G mutation (named pcoSpikeD614G) and define a large-scale production process. According to the in vitro studies, pcoSpikeD614G expressed abundant spike protein in HEK293T cells. After the administration of pcoSpikeD614G to BALB/c mice through intramuscular (IM) route and intradermal route using an electroporation device (ID + EP), it induced high level of anti-S1 IgG and neutralizing antibodies (*P* < 0.0001), strong Th1-biased immune response as shown by IgG2a polarization (*P* < 0.01), increase in IFN-γ levels (*P* < 0.01), and increment in the ratio of IFN-γ secreting CD4^+^ (3.78–10.19%) and CD8^+^ (5.24–12.51%) T cells. Challenging K18-hACE2 transgenic mice showed that pcoSpikeD614G administered through IM and ID + EP routes conferred 90–100% protection and there was no sign of pneumonia. Subsequently, pcoSpikeD614G was evaluated as a promising DNA vaccine candidate and scale-up studies were performed. Accordingly, a large-scale production process was described, including a 36 h fermentation process of *E. coli* DH5α cells containing pcoSpikeD614G resulting in a wet cell weight of 242 g/L and a three-step chromatography for purification of the pcoSpikeD614G DNA vaccine.

## Introduction

Coronaviruses belong to a broad family of RNA viruses and cause respiratory, gastrointestinal, and neurological infections in humans and animals. Among seven types of coronaviruses known to infect humans, the first four strains (HCoV-229E, HCoV-OC43, HCoV-NL63, and HCoV-HKU1) caused only mild symptoms, and the remaining caused serious outbreaks in the twenty-first century which were severe acute respiratory syndrome coronavirus (SARS-CoV) in 2003, Middle East respiratory syndrome coronavirus (MERS-CoV) in 2012, and severe acute respiratory syndrome coronavirus 2 (SARS-CoV-2) in late 2019^1–3^. After the identification of SARS-CoV-2 as a global pandemic, millions of people died globally and a considerable amount of the patients that recovered developed post-acute sequelae of SARS-CoV-2 infection (PASC or Long COVID) or multisystem inflammatory syndrome detected in children (MIS-C)^4,5^. Vaccines have reduced the outcomes of COVID-19 and its complications, however studies showed that vaccine effectiveness decreased over time^6^. Specifically, the effectiveness of mRNA or adenoviral vaccines such as BNT162b2 (Pfizer–BioNTech), mRNA-1273 (Moderna), ChAdOx1 nCoV-19 (AZD1222; Oxford–AstraZeneca), and Ad26.COV2.S (Janssen), has reduced from 83% at 14–42 days to 62% by 112–139 days^7^. SARS-CoV is believed originated from a wildlife trade in Shunde, Guangdong, China, and looking at MERS-CoV, the virus was transmitted from bats to dromedary camel and then from dromedaries to humans. Based on the zoonotic potential of the coronaviruses, future outbreaks are inevitable^8^. Overall, vaccine development studies against SARS-CoV-2 and its variants of concern (VOC) have utmost importance to get ready for future coronavirus outbreaks.

DNA vaccines are among the next-generation vaccine platforms which have potential to accelerate the production of effective vaccines when urgently needed due to their ability to elicit long term both the humoral and cellular immune responses, easy manufacturability, high stability and long-term storability even at room temperature^9,10^. The safety and efficacy of DNA vaccines have also been documented in many clinical trials^11–14^. Particularly, DNA vaccines circumvent concerns associated with other vaccines, such as safety risks being associated with replicating microorganisms or with the use of any viral vectors causing anti-vector immunity and risks linked to the manufacture of inactivated virus vaccines^10,15^. According to the WHO’s landscape of vaccine candidates list as of March 30th, 2023, there are 17 DNA vaccine candidates in various phases of clinical development and 16 DNA vaccine candidates in various phases of pre-clinical development^16^.

In the present study, we developed a DNA vaccine against the SARS-CoV-2 expressing Spike protein with D614G mutation. To determine the Spike protein sequence to be used as antigen, we first identified the dominant circulating variant by screening clinical samples of COVID-19 patients and identified mutations in Turkey by sequencing the Spike gene in these samples. The sequence of the SARS-CoV-2 variant used in this study is not the current VOC because at the time we started this study (June 2020), the VOC was the strain with the D614G mutation. We used sequencing data and in silico methods to design our Spike antigen and then docked our novel Spike antigen with the human ACE2 (Angiotensin Converting Enzyme-2) receptor to determine the binding energy. After the construction of the DNA vaccine, we transfected HEK293T cells and analyzed the protein expression capability by immunofluorescence antibody test (IFAT), Western blot, and Real Time Quantitative PCR (RT-qPCR). Next, we immunized BALB/c mice and K18-hACE2 transgenic mice thrice at days 0, 14 and 56 with the DNA vaccine administered through intramuscular (IM) route and intradermal (ID) route using an electroporator device. We analyzed humoral and cellular immune responses afterwards using Enzyme-Linked Immunosorbent Assay (ELISA), Western blot, surrogate virus neutralization assay, microneutralization assay, Cytokine ELISA, and flow cytometry. After challenging of K18-hACE2 transgenic mice with SARS-CoV-2, gross pathology, histopathological examination, and RT-qPCR were performed. After detecting high immunogenicity and protection levels, large-scale upstream and downstream process optimization studies were conducted.

## Methods

### Determination of circulating SARS-CoV-2 variant to design the vaccine antigen

**Figure 1:**
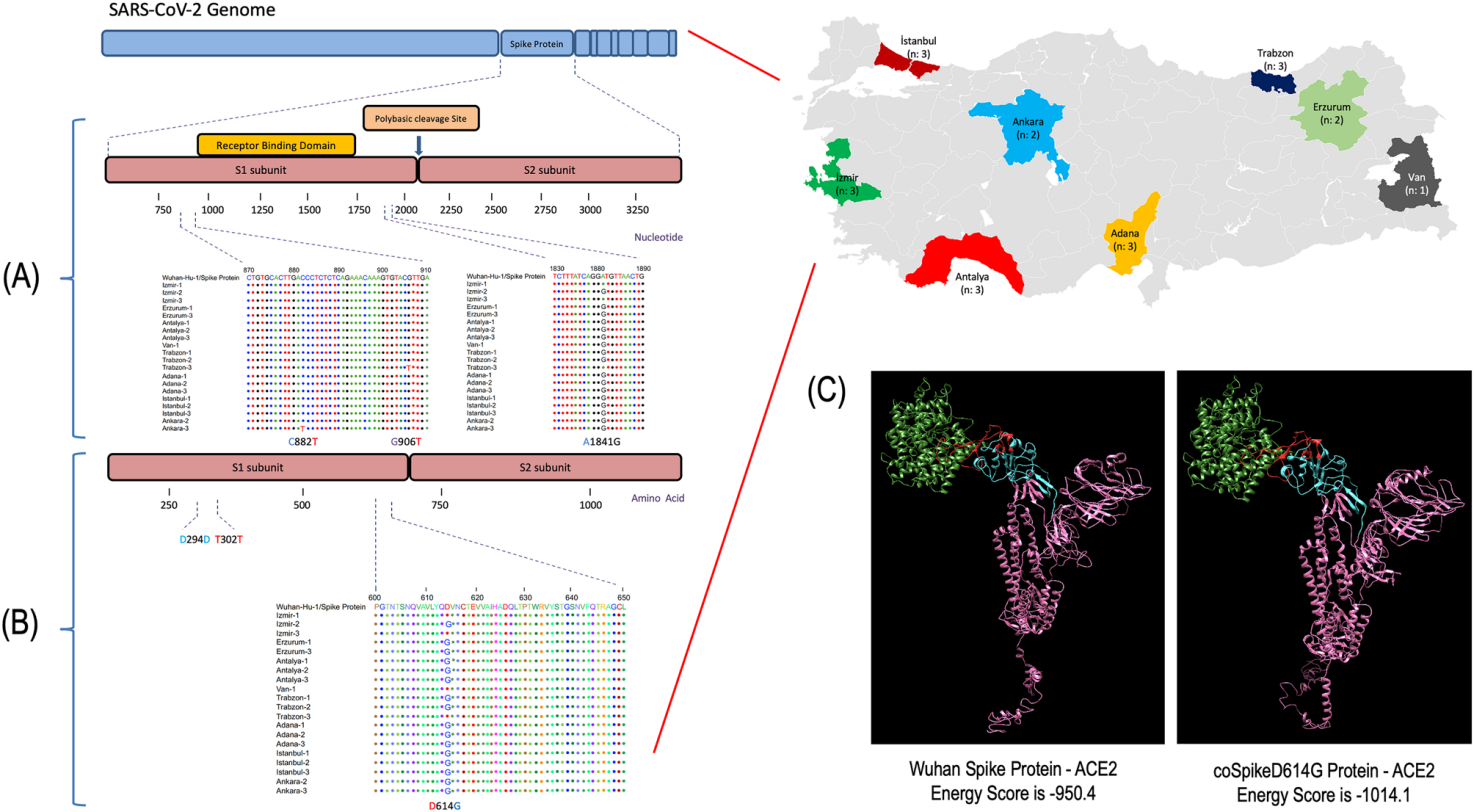
In silico analyses of vaccine antigen. (**A**) Variations detected in *Spike* gene of 20 SARS-CoV-2 isolates collected from various provinces of Türkiye. The map in figure was generated by Microsoft 365 Excel. (**B**) A1841G mutation causes D614G amino acid alteration (**C**) 3D image of human ACE2 receptor docked with Wuhan Spike protein and coSpikeD614G vaccine antigen. Purple color: Spike protein; blue color: receptor binding domain (RBD) of Spike protein; green color: human ACE2 receptor; red color: receptor binding motif of human ACE2 protein.

Before designing the Spike antigen to be used in DNA vaccine, we sequenced the *Spike* gene of SARS-CoV-2 strains (n = 20) isolated from hospitalized patients in seven different provinces of Türkiye (Ankara, Adana, Antalya, İstanbul, İzmir, Trabzon, Erzurum, and Van provinces) in June 2020 (Fig. 1). The RNA samples of these patients were provided by the National Virology Reference Central Laboratory (General Directorate of Public Health, Ministry of Health, Türkiye) and cDNA was synthesized using the Superscript III First-Strand Synthesis System kit (Thermo Fisher Scientific, USA) according to the manufacturer's instructions. For sequencing, the *Spike* gene of Wuhan isolate (Genbank Accession no: NC_045512.2) was divided to into seven fragments and each fragment was amplified by different primer pairs (S1 Table). The PCR mixture was prepared in 25 µl volume containing 2 µl template cDNA, 12.5 µl Dream Taq master mix (Thermo Fisher Scientific, USA), 1 µl of each primer (10 pmol), and 8.5 µl distilled water. The PCR amplifications were carried out under the following conditions: 2 min initial denaturation step at 95 °C, followed by 35 cycles of 1 min at 95 °C, 45 s at 58 °C, and 45 s at 72 °C, and a final extension of 10 min at 72 °C. PCR products were visualized by 1% agarose gel electrophoresis. Then, PCR products were purified by Qiaquick PCR Purification Kit (Qiagen, USA) and sequenced to determine the circulating variant. Generated sequence data was aligned in MEGA 7.0 and compared with Wuhan isolate to reveal variations.

### In silico modeling of spike protein and vaccine antigen design.

3D structural models of SARS-CoV-2 Spike protein were constructed by I-TASSER Server (http://zhanglab.ccmb.med.umich.edu/I-TASSER)^17^ and obtained models were refined by 3Drefine (http://sysbio.rnet.missouri.edu/3Drefine/) using RWplus model analysis^18^. Refined models were evaluated by ProSA-web tool (https://prosa.services.came.sbg.ac.at/prosa.php) for the structural validation analysis^19,20^. Refined and validated 3D models were visualized and compared on UCSF Chimera 1.14 tool^21^. All docking analyses of Spike protein models with Native Human Angiotensin Converting Enzyme-Related Carboxypeptidase (ACE2) (RCSB PDB ID no: 1R42) were performed by ClusPro Server (https://cluspro.bu.edu/home.php)^22^ and visualized on UCSF Chimera 1.14 tool.

### Construction of DNA vaccine

After the analyses of sequence data, the gene encoding Spike protein, which contains a D614G mutation, was codon optimized for expression in *Homo sapiens* (named as coSpikeD614G) and synthetically produced in a pEX-A258/coSpikeD614G vector (Eurofins, Luxembourg). To secrete spike protein outside the host cell, a signal peptide of *Homo sapiens* Ig heavy chain epsilon-1 (V-D-J region) (IGHE) gene (GenBank accession no: AH005278.2; between 4 and 54 bp; size of the fragment 51 bp) was included in frame to 5′- end of the coSpikeD614G (S2 Table). Moreover, a Kozak sequence (in frame to 5′- end), and *NheI* (5′-end) and *XbaI* (3′-end) restriction enzyme sites were included in the design to enable cloning into linearized and dephosphorylated pVAX1 vector (ThermoFisher Scientific, USA). After cloning coSpikeD614G into pVAX1 vector, the resulting DNA vaccine plasmid was named pcoSpikeD614G expressing Spike protein with the theoretical MW of 143.1 kDa. Plasmids were transformed into chemically competent *E. coli* DH5α cells (ThermoFisher Scientific, USA) and grown overnight in LB medium supplemented with kanamycin. Positive colonies were confirmed by double digestion [using *NheI* (NEB, USA) and *XbaI* (NEB, USA)] and with sequencing (Forward primer: 5-GACGTCAATGGGAGTTTGTTTT-3 and reverse primer: 5-ATAGAATGACACCTACTCAGACA-3). Master cell bank and working cell bank samples prepared from glycerol stocks of overnight culture were kept in -80 °C freezer. The DNA vaccine and control (empty plasmid) were produced by inoculating glycerol stocks in LB medium supplemented with kanamycin and purified using Purelink HiPure Expi Plasmid Megaprep Kit according to the manufacturer’s protocol (ThermoFisher Scientific, USA).

### In vitro transfection of HEK293T cells

To evaluate the protein expression level of pcoSpikeD614G, human embryonic kidney cells (HEK293T, ATCC CRL-3216) were cultured on six-well plate (Nunc, USA) and 4-well slides (Nunc, USA), with an initial density of 1 × 10^6^ and 2 × 10^5^ cells per well, respectively^23^. HEK293T cells were transfected with 2.5 µg pcoSpikeD614G /well for six-well plate and 0.5 µg pcoSpikeD614G/well for 4-well slides using Lipofectamine 3000 (ThermoFisher Scientific, USA) reagents according to the manufacturer's instructions. 48 h after the transfection, cells were collected from 6-well plate, lysed with RIPA buffer (ThermoFisher Scientific, USA) and the presence of expressed proteins was determined by Western blot as described in Sect. 2.10. In the second group of 6-well plates, mRNA expression levels of coSpike614G protein were assessed by RT-qPCR as described in Sect. 2.7. In addition, 4-well slides were used in IFAT to show the presence of expressed Spike protein. pVAX1 without an insert was used as negative control.

### IFAT

To show the presence of coSpikeD614G protein expression, transfected HEK293T cells in 4-well slides were fixed with methanol for 5 min and subsequently with cold acetone for 30 s. Then the slides were washed with 1 × PBS for 5 min, permeabilized with 0.1% Triton-X for 15 min, washed again with 1 × PBS for 5 min, and blocked with 1% BSA in 1 × PBS for 30 min at RT. Next, the cells were probed with the mouse sera (obtained from mice vaccinated with pcoSpikeD614G) with a dilution of 1:50 for 1 h at RT, washed 3 times for 5 min and then stained with anti-mouse IgG antibody conjugated with FITC (Sigma Aldrich, USA) with a dilution of 1:100 for 1 h at RT. Thereafter, slides were washed with 1 × PBS for 5 min, mounted with DAPI Fluoromount-G (SouthernBiotech, USA) overnight, and protein expression was visualized using a fluorescence microscope (Nikon, Japan).

### RT-qPCR

To determine the mRNA level of *Spike* gene expressed by pcoSpikeD614G, total RNA was extracted from transfected HEK293T cells in 6-well plates using RNeasy Mini Kit (Qiagen, USA) according to the manufacturer’s instructions. RNA concentrations were determined by Nanodrop (ND-1000, ThermoFisher Scientific, USA). Reverse transcription reaction was performed by SuperScript™ III First-Strand Synthesis System (ThermoFisher Scientific, USA) according manufacturer’s protocol using100 ng of each RNA sample and oligoDT primer provided by the kit. The RT-qPCR amplification was carried out using cDNA and specific codon optimized *Spike* gene primers (Sybr forward 5-CTGCACCCAGCTGAATAGA-3; Sybr reverse 5-AATTGAAGCCGCCGAAGTCC-3) and *β-actin* gene primers (β-actin forward 5-GTGACGTGGACATCCGTAAA-3; β-actin reverse 5-CAGGGCAGTAATCTCCTTCTG-3). The PCR reaction included 1 µl cDNA, 1 µM primer from each and 5 µl LightCycler® 480 SYBR Green I Master (Roche, Germany) and carried out by 1.5 LightCycler Real Time instrument (Roche, Germany) under the conditions of 5 min at 95 °C followed by 40 cycles of 10 s at 95 °C, 20 s at 60 °C for β-actin primers or 58 °C for spike primers, and 30 s at 72 °C. In addition to the negative transfection control (RNA sample obtained from cells transfected with empty pVAX1), NTC containing distilled water were included as control. LightCycler software, Version 3.5 (Roche, Germany), was used to generate each sample’s threshold cycle (CT), and 2^−ΔΔCt^ analysis was performed to analyze the mRNA expression level of the pcoSpikeD614G plasmid.

### Immunization

The immunogenicity study for the pcoSpikeD614G vaccine was carried out in 6–8 weeks old female BALB/c mice purchased from Kobay Experimental Animals Laboratory (Ankara, Türkiye), while 8–10 weeks old female K18-hACE2 transgenic mice provided by TÜBİTAK MRC were used to determine the protective efficacy of the pcoSpikeD614G vaccine. BALB/c mice (15 mice/group) and K18-ACE2 transgenic mice (10 mice/group) were immunized thrice on days 0, 14, and 56 with pcoSpikeD614G diluted in 1X Dulbecco’s phosphate-buffered saline (ThermoFisher Scientific, USA) under anesthesia [ketamine hydrochloride (Ketalar) 2 mg/kg (1 ml—5 mg) + 2% xylazine (Alfazyne) 3 mg/kg (1 ml—20 mg) as intraperitoneally]. The pcoSpikeD614G vaccine or negative control containing empty pVAX1 plasmid were administered intradermally (ID; 25 µg plasmid/dose) to the lumbar dermis using 12.7 mm x 30G needle using an electroporation (EP) device (AgilePulse, USA) or intramuscularly (IM; 100 µg plasmid/dose) using a 26-gauge needle to the anterior tibial muscle (S3 Table). Before and two weeks after each immunization, blood samples were collected by tail bleeding under anesthesia and sera were separated by centrifugation at 3000 rpm for 10 min and stored at − 20 °C until use.

### ELISA detecting anti-S1 IgG antibodies

The level of anti-S1 IgG antibodies in serum samples was determined by ELISA as described previously with slight modifications^24,25^. Briefly, microtiter plates (Nunc, USA) were coated overnight at 4 °C with 0.5 µg/well recombinant S1 protein containing D614G mutation (Sino Biological, China) diluted in 1 × PBS. The next day, plates were washed thrice with 1 × PBS-T and blocked for 30 min with 0.5% non-fat dry milk in 1 × PBS-T at 37 °C. Then, the plates were washed thrice and probed with 100 μl mouse sera diluted to 1:100 in blocking buffer for 2 h at RT. Next, the plates were washed thrice and probed with anti-mouse IgG (Sigma, USA), IgG1 (Santa Cruz, USA), and IgG2a (Santa Cruz, USA) antibodies conjugated with peroxidase diluted to 1:3000; 1:2500; 1:1500, respectively in 1 × PBS-T for 1 h at RT. Thereafter, 100 μl tetramethylbenzidine substrate solution (ThermoFisher Scientific, USA) was added to each well, and the reaction was stopped with 1N H_2_SO_4_. The results were evaluated in a microplate reader (Bio-Tek EL × 808, USA) at 450 nm.

### SDS-PAGE and Western blot

Recombinant S1 and S1 + S2 proteins (SinoBiological, China) or supernatants of transfected HEK293T cell lysates were separated by 12% Sodium dodecyl sulfate–polyacrylamide gel electrophoresis (SDS-PAGE) and transferred to PVDF membrane (Immobilon-P, Millipore, USA) as described^26^. The membranes were incubated with vaccinated mice sera pools as primary antibody diluted to 1:50 or anti-β actin (diluted to 1:3000, Sigma-Aldrich, USA) or anti poly-His (1:3000, Sigma Aldrich, USA) antibodies for 1.5 h at RT. Then membranes were probed with alkaline phosphatase-conjugated goat anti-mouse IgG antibody (Sigma Aldrich, USA) diluted to 1:2000 for 1 h at RT. Next, the blots were visualized by alkaline phosphatase-developing buffer (0.1 M Na_2_CO_3_, pH 9.5, 0.1 M NaCl, 5 mM MgCl_2_) mixed with 5-bromo-4-chloro-3-indolyl phosphate (BCIP) and nitro blue tetrazolium (NBT) (Applichem, Germany).

### SARS-CoV-2 surrogate virus neutralization test

Neutralizing antibody responses in vaccinated mice sera were analyzed using an ELISA-based SARS-CoV-2 surrogate Virus Neutralization Test (sVNT) which is a S1 protein/ACE2 ligand binding assay) (AffinityImmuno, Canada) according to the manufacturer’s protocol. Initially, the mice sera diluted to 1:10 and calibrators provided by the kit were added to pre-coated wells of the plate. Then, detection reagent was added to each well and the plate was incubated for 1 h at RT. After washing and adding tetramethylbenzidine substrate solution to each well, the reaction was stopped with 1N H_2_SO_4_ and results were evaluated in a microplate reader (Bio-Tek EL × 808, USA) at 450 nm against 620 nm. Neutralizing antibody levels were calculated with a standard curve constructed by plotting the absorbance values obtained from each standard.

### Microneutralization test (MNT)

Vero E6 cells, obtained from American Type Culture Collection (ATCC: CRL-1586) were maintained using Dulbecco’s Modified Eagle’s Medium (DMEM) supplemented with 10% fetal bovine serum and 1% penicillin/streptomycin (ThermoFisher Scientific, USA) in an incubator at 37 °C under 5% CO_2_^27^.

The SARS-CoV-2 virus detected from the combined nasal and throat swab of a COVID-19 patient was grown in Vero E6 cells in the BSL-3 facility located in National Virology Reference Central Laboratory, Ankara, Türkiye. The virus was named as hCoV-19/Türkiye/27/2020 and passaged two times in Vero E6 cells. The stock virus was harvested, aliquoted, and stored at − 80 °C until use.

The microneutralization test (MNT) was performed in a BSL-3 facility located in National Virology Reference Central Laboratory, Ankara, Türkiye. The virus stock was titrated in microtiter plate on Vero E6 cells in serial log_10_ dilutions (dilution factor was 10) to obtain 50% tissue culture infectious dose (TCID_50_). The plates were incubated at 37 °C under 5% CO_2_ for 4 days and observed for cytopathic effect (CPE) daily. The endpoint of viral dilution leading to CPE in 50% of inoculated wells (TCID_50_) was calculated by the Reed-Muench method^28^.

The heat-inactivated vaccinated mouse sera were twofold serially diluted in a microtiter plate starting from 1:4 in DMEM supplemented with 2% FBS. Next, an equal volume of 100 TCID_50_ of the SARS-CoV-2 propagated as described above was added to the serum dilutions and incubated for 1 h at 37 °C under 5% CO_2_. Thereafter, 100 μl Vero E6 cells (2 × 10^5^ cells/ml in DMEM supplemented with 2% FBS) were added to the virus + serum mixture, and plates were incubated for 4 days at 37 °C under 5% CO_2_.

Virus dilution was back titrated by replacing serum with medium in each experiment to determine the virus test dose. Neutralization was assessed by CPE using phase contrast microscopy. The complete inhibition of virus propagation in an individual well was accepted as a positive result. The neutralization endpoint titer was determined as the highest serum dilution that inhibited the virus infection in 50% of the inoculated wells (Virus Neutralization Titer 50-VNT50)^29^. The VNT50 titer ≥ 4 was considered as positive.

### Splenocyte isolation and stimulation

To determine the cellular immune response, spleens of mice (5 mice/group) were removed aseptically two weeks after the third vaccination. Single cell suspensions were prepared as previously described^26^. A total of 5 × 10^5^ viable splenocytes were ex vivo stimulated with the peptide pool described previously^30,31^ (S4 Table) and incubated for 72 h at 37 °C under 5% CO_2_. The cells were treated with concanavalin A (10 μg/ml) (Sigma Aldrich, USA) or cell stimulation cocktail (ThermoFisher Scientific, USA) as positive control and with the medium as negative control.

### Cytokine ELISA

The level of IL-4 and IFN-γ in stimulated splenocyte culture supernatants were determined by ELISA kits according to the manufacturer’s protocol (ThermoFisher Scientific, USA). Briefly, 100 µl/well of the splenocyte supernatants were added to each well of the plates and absorbance values were measured at 450 nm using a microplate reader (Bio-Tek EL × 808, USA). Serially diluted mouse IL-4 and IFN-γ proteins provided by the kit were used to generate standard curves and calculate the level of cytokines in the cell culture supernatants. The limit of detection for IL-4 and IFN-γ were 4 pg/ml and 15 pg/mL respectively.

### Flow cytometry

To evaluate the percentage of IL-4 secreting CD4^+^ T cells as well as IFN-γ secreting CD4^+^ and CD8^+^ T cells, stimulated splenocytes were stained with Alexa flour 647 conjugated anti-CD3 (0.25 µg/well), FITC-conjugated anti-CD8^+^ (0.5 µg/well), PerCP-cyanine 5.5 conjugated anti-CD4^+^ (0.5 µg/well), PE-Cyanine 7 conjugated anti-IL-4 (0.2 µg/well) and PE-conjugated anti-IFN-γ (0.2 µg/well) antibodies (ThermoFisher Scientific, USA). Intracellular fixation and permeabilization buffer set (ThermoFisher Scientific, USA) were used for fixation and permeabilization of splenocytes. Stained cells were analyzed using a BD LSRFortessa™ cell analyzer and BD FACSDiva 8.0.1 software (BD Bioscience, USA).

### Challenge of K18-hACE2 transgenic mice with SARS-CoV-2 virus

Immunized K18-hACE2 transgenic mice were challenged with SARS-CoV-2 virus (hCoV-19/Türkiye/Pen07/2020, EPI_ISL_491476) which was isolated in Pendik Veterinary Control Institute. Two weeks after the third vaccination, mice were challenged with 10^5^ TCID_50_ virus administered intranasally for 3 consecutive days in Animal BSL-3 facility of TÜBİTAK MRC. The mice were checked for clinical symptoms and weighed every day. On the 15th day of instillation, a gross pathological examination of organs was performed. Scoring of the lungs was done to evaluate the pneumonia^32^. Accordingly, healthy lungs without lesions were scored 0, lungs with edema and hyperemia were scored between 0.5 and 1, different rates of pneumonia lesions were scored between 1.5 and 5, and dead mice were scored 5. Lungs were also collected for histopathological examination and determination of virus load by RT-qPCR as previously described^33,34^.

Briefly, RNA was extracted using QIAamp Viral RNA Mini kit (Qiagen, USA) according to the manufacturer’s protocol. Viral RNA detection was performed using One Step PrimeScript III RT‐qPCR Kit (Takara, Japan) according to the manufacturer’s protocol. The RT-qPCR targeting nucleocapsid (NC) gene of SARS‐CoV‐2 was performed as previously described ^34,35^. Briefly, the primer and probe sets (NC1 and NC2) were NC1 Forward: 5′‐GACCCCAAAATCAGCGAAAT‐3′, NC1 Reverse: 5′‐TCTGGTTACTGCCAGTTGAATCTG‐3′, NC1 Probe: 5′‐FAM‐ACCCCGCATTACGTTTGGTGGACC‐BHQ1‐3′. NC2 Forward: 5′‐TTACAAACATTGGCCGCAAA‐3′, NC2 Reverse: 5′‐GCGCGACATTCCGAAGAA‐3′, NC2 Probe: 5′‐FAM‐ACAATTTGCCCCCAGCGCTTCAG‐BHQ1‐3′. All reactions were performed under the following conditions of 52 °C for 5 min, 95 °C for 10 s, followed by 44 cycles of 95 °C for 5 s and 55 °C for 30 s using a CFX96 Touch instrument (BioRad, USA). For histopathological examination, lungs were fixed with 10% buffered formalin for 48–72 h, dehydrated in a series of alcohol solutions of ascending concentration, and embedded in paraffin wax. Sections were cut to a 5 µm thickness and stained with hematoxylin for 5 min and then stained with eosin. The stained sections were dehydrated, cleared, and mounted. The slides were viewed under the Zeiss Axio Vert A1 microscope (Zeiss, Germany) and zen software V2.6 (Zeiss, Germany). Semi-quantitative assessment was used in the study. The inflammation status in the lungs was compared to the whole lung and given a score between 0 and 3. Accordingly, 0 = No inflammation; 1 = Morphology normal but mild erythrocyte and lymphocyte infiltration around the bronchioles; 2 = Moderate erythrocyte and lymphocyte infiltration in the lung; 3 = Impaired morphology, intense erythrocyte and lymphocyte infiltration in the lung. Lungs of dead mice were not examined.

### Large-scale production of pcoSpikeD614G DNA vaccine

Large-scale upstream and downstream process studies were described, including a fermentation process of *E. coli* DH5α cells containing pcoSpikeD614G and a three-step chromatography for purification of the pcoSpikeD614G DNA vaccine. Briefly, 1 ml of frozen working cell bank sample was inoculated to 400 ml of terrific broth supplemented with glycerol (4 ml/L) and incubated at 30 °C, 200 rpm overnight. *E. coli* DH5α cells containing pcoSpikeD614G DNA vaccine were cultured for 36 h in a modified chemically defined medium^36^ using a 10 L vessel of bioreactor (New Brunswick BioFlo115, USA). The initial fermentation medium was prepared as follows: 475 mL of 10 × phosphate/citric acid buffer (133 g/L KH_2_PO_4_, 40 g/L (NH_4_)_2_HPO_4_, 17 g/L citric acid) and 3.763 L distilled water were added to the 10 L vessel and autoclaved at 121 °C for 20 min. After the medium was cooled to RT, pH of the medium was adjusted to 6.8 with 25% ammonium hydroxide, and the following sterile solutions were added aseptically to the vessel to make the complete fermentation medium: 12.5 ml of 240 g/L MgSO_4_, 1.1 ml of 20 g/L thiamine solution, 48 ml of 100 × trace element solution, 50 µg/ml kanamycin, and 25 ml of 70% glucose solution. The 100 × trace element solution contained: 10 g/L iron (III) citrate, 0.25 g/L CoCl_2_.6H_2_O, 1.5 g/L MnCl_2_.4H_2_O, 0.105 g/L CuCl_2_.2H_2_O, 0.3 g/L H_3_BO_3_, 0.25 g/L Na_2_MoO_4_.2H_2_O, 1.3 g/L zinc acetate.2H_2_O, 0.84 g/L EDTA. The feeding medium was prepared by mixing 40 mL of 240 g/L MgSO_4_, 2 ml of 20 g/L thiamine solution, 18 ml of 100 × trace element solution, and 540 ml of 70% glucose solution. After adding overnight culture to the vessel, the pH was adjusted to $\sim$ 7 with 25% ammonium hydroxide throughout the fermentation. Antifoam 204 (Sigma-Aldrich, USA) was added between 24 and 36 h of fermentation, as foam accumulation warranted (final concentration < 0.01%). Cell growth rate and glucose concentration were monitored by OD_600_ (Bio-Tek EL × 808, USA) and glucometer (Roche, Germany). Feeding was initiated as glucose concentration went below 2 g/L and glucose levels were maintained below 2 g/L thereafter. Dissolved oxygen level (pO_2_) was kept between 32 and 36. Agit was kept between 400 and 800 rpm depending on the level of pO_2_. At the 36th hour of cultivation, the culture medium was collected and centrifuged at 5000 × g for 10 min. Thereafter, downstream processing was performed using two main steps which are alkaline lysis and column chromatography. For alkaline lysis, every 1 g of *E. coli* DH5α cell pellet was resuspended with 10 ml of resuspension buffer (50 mM Tris–HCl, 10 mM EDTA, pH: 8) and mixed with 10 ml of lysis buffer (0.2 M NaOH containing %1 SDS) and incubated for 5 min. Next, 10 ml of neutralizing buffer (3 M potassium acetate, pH: 5,5) was added to the mixture and centrifuged at 20,000 × g for 20 min. Supernatants were collected and passed through 0.45 µm bottle top filter. Column chromatography was performed with the filtered and cleared supernatant. A three-step purification was performed as previously described^37^ using ÄKTA Fast protein liquid chromatography (FPLC) and Unicorn software program. In the first step, size exclusion column (Sepharose 6 fast flow, Cytiva, USA) was used to remove RNA from the DNA sample using 2.1 M (NH_4_)_2_SO_4_, 10 mM EDTA, 100 mM Tris–HCl, pH 7.5 buffer. In the second step of purification, supercoiled plasmid DNA is dissociated from open circular plasmid DNA by the hydrophobic interaction column (Captoplasmid, Cytiva, USA) using 2.0 M (NH_4_)_2_SO_4_, 10 mM EDTA, 100 mM Tris–HCl, pH 7.5 as binding buffer and 0.3 M NaCl, 1.7 M (NH_4_)_2_SO_4_, 10 mM EDTA, 100 mM Tris–HCl, pH 7.5 as elution buffer. In the third step of purification, the supercoiled plasmid DNA is polished by the anion exchanger column (HiTrap SOURCE 30Q, Cytiva, USA) using 0.4 M NaCl, 10 mM EDTA, 100 mM Tris–HCl, pH 7.5 as binding buffer and 1.0 M NaCl, 10 mM EDTA, 100 mM Tris–HCl, pH 7.5 as elution buffer. The final purified plasmid DNA was mixed with 0.7 volume isopropanol and centrifuged at 30,000 × g for 30 min. At the end of centrifugation, the supernatant was removed, the pellet was resuspended with 5 ml 70% ethanol and centrifuged at 30,000 × g for 10 min. Thereafter, the supernatant was removed, and the pellet was resuspended with 1 × PBS and kept at − 20 °C. The final product was visualized with 1% agarose gel electrophoresis.

### Statistical analyses

The data's normality was examined, and the normal distribution was validated by column analysis using GraphPad Prism's normality and lognormality. Prism 10 software (GraphPad, USA) was used for the statistical analyses, which included unpaired t-test, Mann–Whitney test, and Kaplan–Meier survival analysis. The data were considered significant if *P* < 0.05. The data in all parametric tests were presented as mean ± standard deviation (SD), whereas data for the non-parametric test (Microneutralization assay) were presented as geometric mean titer (GMT) ± standard deviation (SD).

### Ethical approval

Nasopharyngeal swab samples were collected from humans in June 2020 to determine the circulating SARS-CoV-2 variant of concern. This study was approved by the Local Research Ethics Committee of the Ege University, Faculty of Medicine (Protocol # 20–5.1 T/25). All participants were provided with written informed consent. Data privacy protection was guaranteed by the anonymization of samples. All methods were performed in accordance with the relevant guidelines and regulations. All animal experiments were performed under the instructions and approval of the Institutional Animal Care and Use Committee (IACUC) of Ege University and TÜBİTAK for animal ethical norms (Permit numbers: 2020–083 and 16,563,500–111-3025). All methods used in the study were carried out in accordance with the relevant directives and regulations. All experimental studies were carried out in accordance with ARRIVE guidelines. The challenge of K18-ACE2 transgenic mice with virulent SARS-CoV-2 strain was performed at the Scientific and Technological Research Council of Türkiye, Marmara Research Center, Vice Presidency of Life Sciences, Medical Biotechnology Unit (TÜBİTAK MRC). The experiments were carried out in Biosafety Level 3 (BSL3) and animal BSL3 (ABSL3) facilities at TÜBİTAK MRC.

## Results

### Designing vaccine antigen by sequencing data and in silico analyses

During the first months of the pandemic, a total of 20 clinical samples collected from COVID-19 patients living in 8 different provinces of Türkiye were analyzed for the presence of mutations introduced to *Spike* gene of SARS-CoV-2. According to the results, three different mutations were detected which were C882T (n = 1), G906T (n = 1), and A1841G (n = 17) (Fig. 1A). Among these mutations, C882T and G906T were silent mutations while A1841G mutation caused D614G alteration in the amino acid sequence of 17 isolates (Fig. 1B). Since 85% (17/20) of the isolates contained A1841G mutation, the Spike protein containing D614G variation was used as vaccine antigen (named coSpikeD614G). The coSpikeD614G and Spike protein of Wuhan isolate were docked with the human ACE2 receptor and docking results showed a lower energy score (-1014.1 energy score) between coSpikeD614G and human ACE2 receptor compared to Spike of Wuhan isolate (-950.4 energy score), indicating a stronger binding affinity between coSpikeD614G protein and human ACE2 receptor (Fig. 1C).

### Construction of pcoSpikeD614G DNA vaccine and its expression profile in HEK293T cells

**Figure 2:**
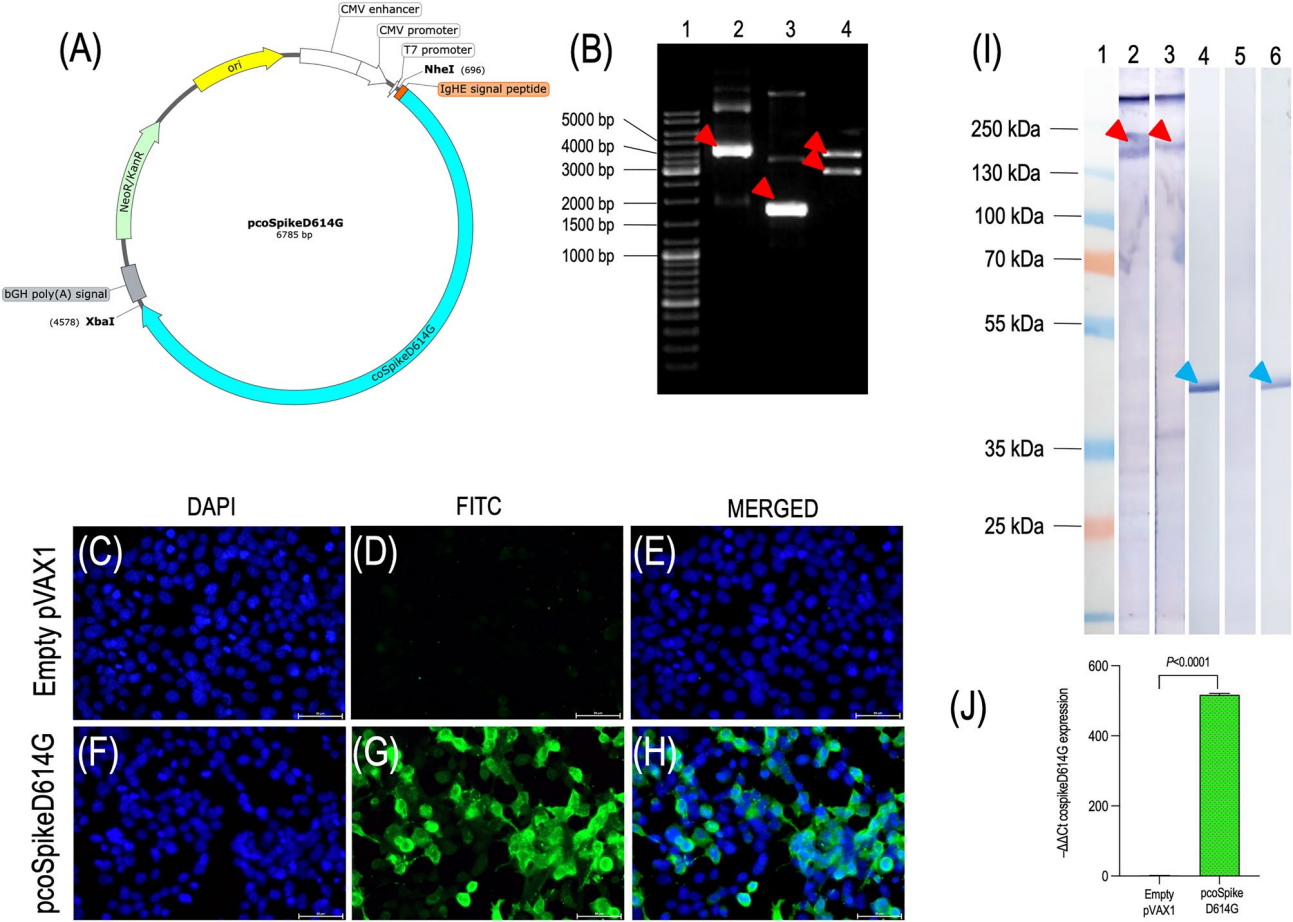
Construction of pcoSpikeD614G DNA vaccine and in vitro expression in HEK293T cells. (**A**) The design pcoSpikeD614G plasmid with size of 6785 bp as shown by SnapGene (**B**) Agarose gel image showing double digestion of pcoSpikeD614G plasmid. Lane 1: DNA ladder; Lane 2: Circular pcoSpikeD614G (red arrowhead); Lane 3: circular empty pVAX1 vector (read arrowhead); Lane 4: double digested pcoSpikeD614G plasmid, upper red arrowhead represents coSpikeD614G gene fused in frame with IGHE signal peptide (3878 bp) and lower red arrowhead represents linearized pVAX1 plasmid (2907 bp). The original pVAX1 plasmid has a size of 2999 bp and removing multiple cloning site with restriction enzymes lowers the size to 2907 bp. (**C–H**) IFAT images showing the in vitro transfection of HEK293T cells with pcoSpikeD614G and empty pVAX1 plasmid. Scale bars represent 50 µm. (**C** and **F)** show DAPI stained nuclei of cells transfected with empty pVAX1 plasmid and pcoSpikeD614G. As shown in (**D/E** and **G/H**), coSpikeD614G protein expression does not exist in pVAX1 transfected cells but is abundantly expressed in pcoSpikeD614G transfected cells. **(I)** Western blot image shows the presence of coSpikeD614G protein expression in HEK293T cell lysates. Lane 1: Protein ladder; Lanes 2 and 3: lysate of pcoSpikeD614G transfected cells probed with pooled vaccinated mice sera immunized thrice through ID + EP and IM routes. Red arrowheads show the recombinant pcoSpikeD614G proteins ($\sim$ 143 kDa); Lane 5: lysate of empty pVAX1 transfected cells probed with pooled vaccinated mice sera immunized with empty pVAX1. Lanes 4 and 6 represent pcoSpikeD614G and empty pVAX1 transfected cell lysate probed with anti β-actin antibody. Blue arrowheads show the expression of β-actin ($\sim$ 42 kDa). Original Western blot images have been shown in Supplementary file named S5 Figure. (**J**) RT-qPCR results show a significant level of coSpikeD614G transgene expression.

According to the results of in silico analyses, the prevalent SARS-CoV-2 variant contained D614G alteration in Spike gene, there was a higher binding affinity between Spike protein with D614G to ACE2 receptor compared to Wuhan strain in docking analyses. For these reasons DNA vaccine was constructed by inserting SARS-CoV-2 Spike protein containing D614G alteration (coSpikeD614G) which is codon optimized for *Homo sapiens* fused in frame with IGHE signal peptide to its N-terminus to pVAX1 plasmid and named pcoSpikeD614G (Fig. 2A). Double digestion results confirmed the insertion of coSpikeD614G gene into pVAX1 plasmid (Fig. 2B). To show the protein expression, cell culture lysates obtained from in vitro transfection of HEK293T cells with pcoSpikeD614G and empty pVAX1 plasmid were analyzed by IFAT, Western blot and RT-qPCR assays. IFAT images showed that pcoSpikeD614G transfected HEK293T cells express recombinant coSpikeD614G protein (Fig. 2C–H). Western blot results verified the expression of recombinant coSpikeD614G protein with a molecular weight of over $\sim$ 143 kDa due to glycosylation (Fig. 2I). The RNA expression level of the coSpikeD614G gene in HEK293T cells transfected with pcoSpikeD614G was measured using total RNA extracted from the transfected HEK293T cells, and RT-qPCR confirmed a significant level of coSpikeD614G transgene expression compared to control transfected with empty pVAX1 plasmid (*P* < 0.0001) (Fig. 2J).

### Humoral immune response

**Figure 3:**
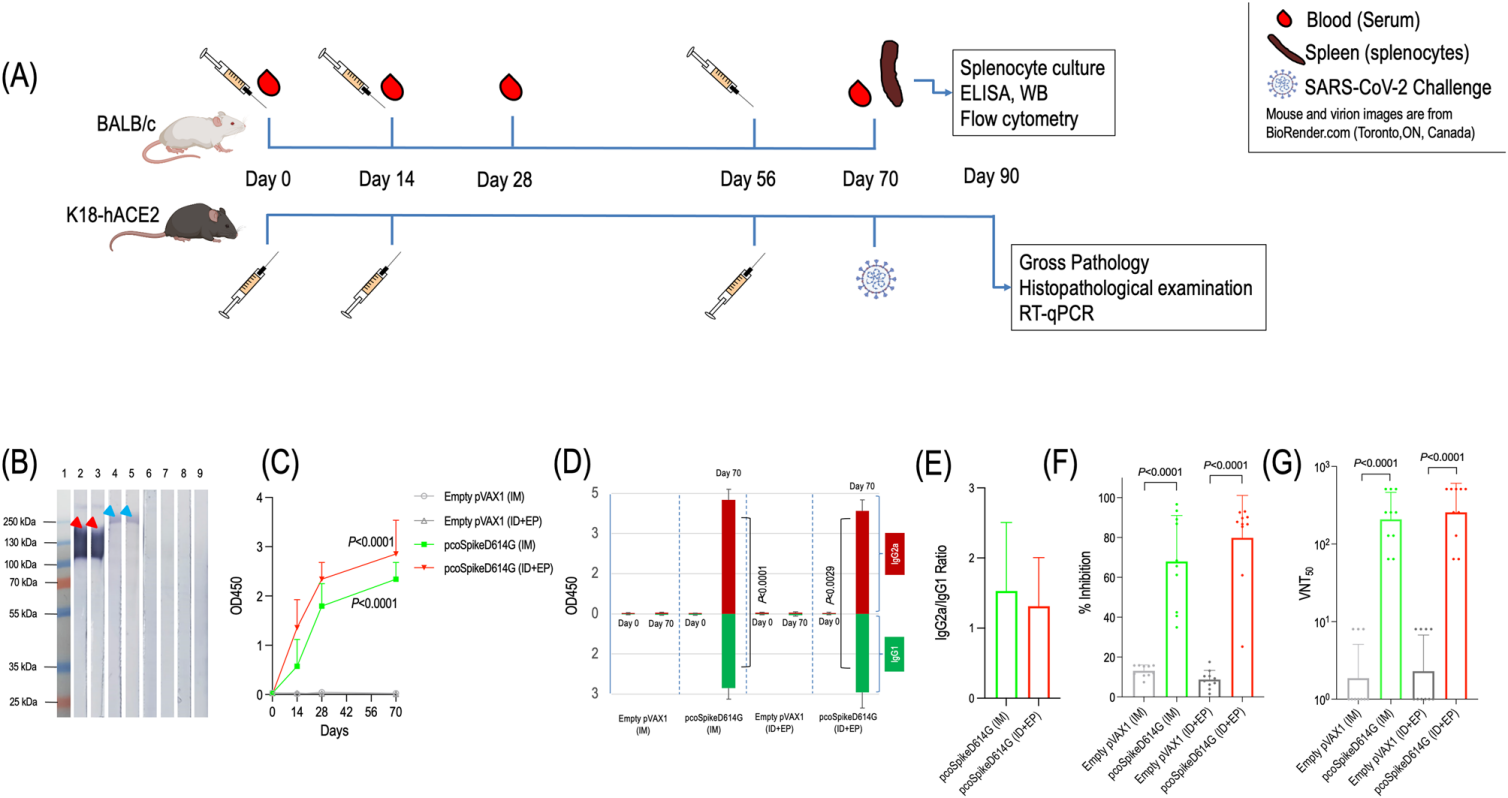
Animal studies and humoral immune response elicited by pcoSpikeD614G DNA vaccine. (**A**) Brief timeline of animal studies. BALB/c mice were immunized to assess the immunogenicity and K18-hACE2 transgenic mice were immunized to determine protective efficacy conferred by pcoSpikeD614G DNA vaccine (**B**) Western blot image shows the presence of anti-S1 and anti S1 + S2 antibody responses in sera of vaccinated BALB/c mice. Lane 1: Protein ladder; Lanes 2 and 3: recombinant S1 protein probed with pooled pcoSpikeD614G vaccinated mice sera immunized thrice through ID + EP and IM routes. Red arrowheads show the recombinant S1 proteins with a size of over $\sim$ 76.41 kDa due to glycosylation; Lanes 4 and 5: recombinant S1 + S2 protein pooled with pcoSpikeD614G vaccinated mice sera immunized thrice through ID + EP and IM routes. Blue arrowheads show the recombinant S1 + S2 proteins with a size of over $\sim$ 138.5 kDa due to glycosylation; Lane 6–7 and 8–9: recombinant S1 and S1 + S2 proteins probed with pooled vaccinated mice sera administered with empty pVAX1. Original Western blot images have been shown in Supplementary file named S5 Figure (**C**) The anti-S1 IgG kinetics derived from mice immunized with pcoSpikeD614G administered through ID + EP and IM routes show significant increase at day 70 compared to controls (**D**) IgG2a/IgG1 polarization was assessed at days 0 and 70 for empty pVAX1 and pcoSpikeD614G and IgG2a responses were significantly higher (**E**) The ratio of IgG2a/IgG1 is slightly higher in pcoSpikeD614G administered through IM route (**F**) SARS-CoV-2 Surrogate Virus Neutralization Test showed that inhibition potential of 1:10 diluted sera obtained from vaccinated mice at day 70 was significantly higher than controls (**G**) SARS-CoV-2 50% neutralization titers (VNT50) in sera of in pcoSpikeD614G immunized mice collated on day 70. Data are presented as GMT ± SD.

The humoral immune response induced by administering pcoSpikeD614G vaccine to BALB/c mice through IM and ID + EP routes was assessed by Western blot, ELISA, ELISA-based SARS-CoV-2 Surrogate Virus Neutralization Test, and Microneutralization test (MNT) (Fig. 3A). Western blot analyses of pcoSpikeD614G vaccinated mice sera diluted to 1:50 and collected at day 70 (two weeks after the third vaccination) showed strong anti-S1 and anti-S1 + S2 IgG antibody responses (Fig. 3B). To quantify and assess the kinetics of anti-S1 IgG response as well as to determine IgG2a/IgG1 polarization, ELISA was performed with 1:100 diluted mice sera collected at day 0 and two weeks after each vaccination (days 14, 28, and 70). Accordingly, pcoSpikeD614G administered through ID + EP or IM routes induced significantly higher anti-S1 IgG responses and increased after each vaccination compared to controls (*P* < 0.0001) while empty pVAX1 vaccinated mice did not induce any anti-S1 IgG response administered both ways (Fig. 3C). Analyses of IgG2a/IgG1 polarization showed that significant IgG2a responses were induced by pcoSpikeD614G administered through ID + EP (*P* = 0.0029) or IM (*P* < 0.0001) routes compared to IgG1 responses at day 70 indicating Th1 biased immune response in each vaccination (Fig. 3D). In addition, at day 70 the IgG2a/IgG1 ratio was slightly higher in mice group pcoSpikeD614G administered through IM route compared to ID + EP route (Fig. 3E). Results of SARS-CoV-2 Surrogate Virus Neutralization Test showed that even the 1:10 diluted sera collected at day 70 from mice administered with pcoSpikeD614G through ID + EP and IM routes have significantly higher inhibition rates compared to controls (*P* < 0.0001) (Fig. 3F). In the microneutralization assay, the inhibitory potential of pcoSpikeD614G was evaluated against live SARS-CoV-2 virus. The results showed that geometric mean titers (GMTs) of VNT50 values obtained from sera of pcoSpikeD614G administered through ID + EP and IM routes were 207.9 and 256 and significantly higher than controls (*P* < 0.0001) (Fig. 3G).

### Cellular immune response

**Figure 4:**
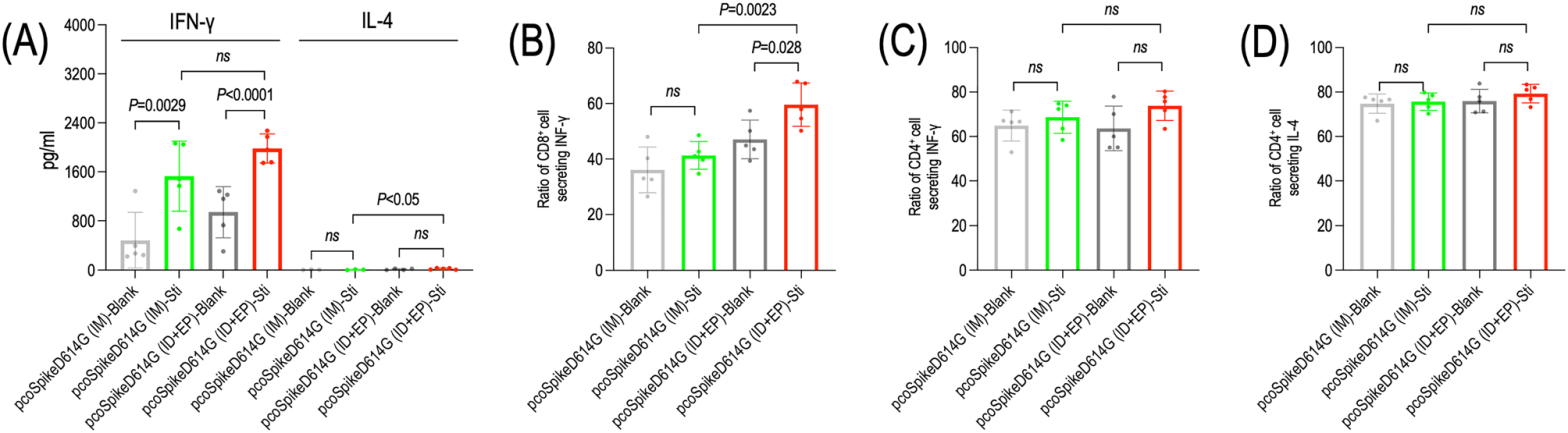
Cellular immune response elicited by pcoSpikeD614G DNA vaccine. (**A**) Cytokine levels were calculated by ELISA from supernatants of cultured splenocytes stimulated with peptide pool. IFN-γ response elicited by pcoSpikeD614G administered through ID + EP and IM routes were increased by 3.15 and 2.1 times, respectively (**B**) The ratio of CD8^+^ cells secreting IFN-γ measured by flowcytometry from cultured splenocytes stimulated with peptide pool. The highest increase in the ratio of CD8^+^ cells secreting IFN-γ was achieved in the mice group immunized by pcoSpikeD614G administered through ID + EP route. The results represent the ratio of CD8^+^ cells secreting IFN-γ over total CD8^+^ cells (**C**) The ratio of CD4^+^ cells secreting IFN-γ measured by flowcytometry from cultured splenocytes stimulated with peptide pool. The highest increase in the ratio of CD4^+^ cells secreting IFN-γ was achieved in the mice group immunized by pcoSpikeD614G administered through ID + EP route. The results represent the ratio of CD4^+^ cells secreting IFN-γ over total CD4^+^ cells (**D**) The ratio of CD4^+^ cells secreting IL-4 measured by flowcytometry from cultured splenocytes stimulated with peptide pool. The highest increase in the ratio of CD4^+^ cells secreting IL-4 was achieved in the mice group immunized by pcoSpikeD614G administered through ID + EP route. The results represent the ratio of CD4^+^ cells secreting IL-4 over total CD4^+^ cells.

The cellular immune response elicited by pcoSpikeD614G vaccine in BALB/c mice administered through IM and ID + EP routes was assessed by cytokine ELISA and flow cytometry. Extracellular cytokine levels calculated from supernatants of cultured splenocytes stimulated by peptide pool for 72 h showed that the mean IFN-γ levels of mice administered with pcoSpikeD614G through IM and ID + EP routes were 1528.12 pg/ml and 1981.25 pg/ml and significantly higher than unstimulated cells (*P* = 0.0029; *P* < 0.0001). There was not a significant difference between IFN-γ levels of mice administered with pcoSpikeD614G through IM and ID + EP routes. The mean IL-4 levels of mice administered with pcoSpikeD614G through IM and ID + EP routes were 3.87 pg/ml and 21.07 pg/ml and the difference between unstimulated cells was not significant (Fig. 4A).

To determine the ratios of CD8^+^ and CD4^+^ cells secreting IFN-γ and CD4^+^ cells secreting IL-4, flow cytometry analyses were performed with cultured splenocytes stimulated by peptide pool for 72 h. Accordingly, the ratio of CD8^+^ cells secreting IFN-γ increased in both mice groups immunized with pcoSpikeD614G administered through IM and ID + EP routes. In the mice group immunized with pcoSpikeD614G administered through IM route, the mean ratio of CD8^+^ cells secreting IFN-γ increased by %5.24 however this increase was not statistically significant. In the mice group immunized with pcoSpikeD614G administered through ID + EP route, the mean ratio of CD8^+^ cells secreting IFN-γ increased significantly by %12.51 (*P* = 0.028). Moreover, the ratio of CD8^+^ cells secreting IFN-γ in mice group immunized with pcoSpikeD614G administered through ID + EP route was significantly higher than mice group immunized with pcoSpikeD614G administered through IM route (*P* < 0.0023) (Fig. 4B). The ratio of CD4^+^ cells secreting IFN-γ also increased in both mice groups immunized with pcoSpikeD614G administered through IM and ID + EP routes. In the mice group immunized with pcoSpikeD614G administered through IM route and ID + EP route, the mean ratios of CD4^+^ cells secreting IFN-γ increased by %3.78. and %10.19 in comparison with unstimulated cells, respectively (Fig. 4C). The ratio of CD4^+^ cells secreting IL-4 increased by %0.91 and %3.34 in both mice groups immunized with pcoSpikeD614G administered through IM and ID + EP routes (Fig. 4D).

### Challenge of K18-hACE2 transgenic mice with SARS-CoV-2 virus results

**Figure 5:**
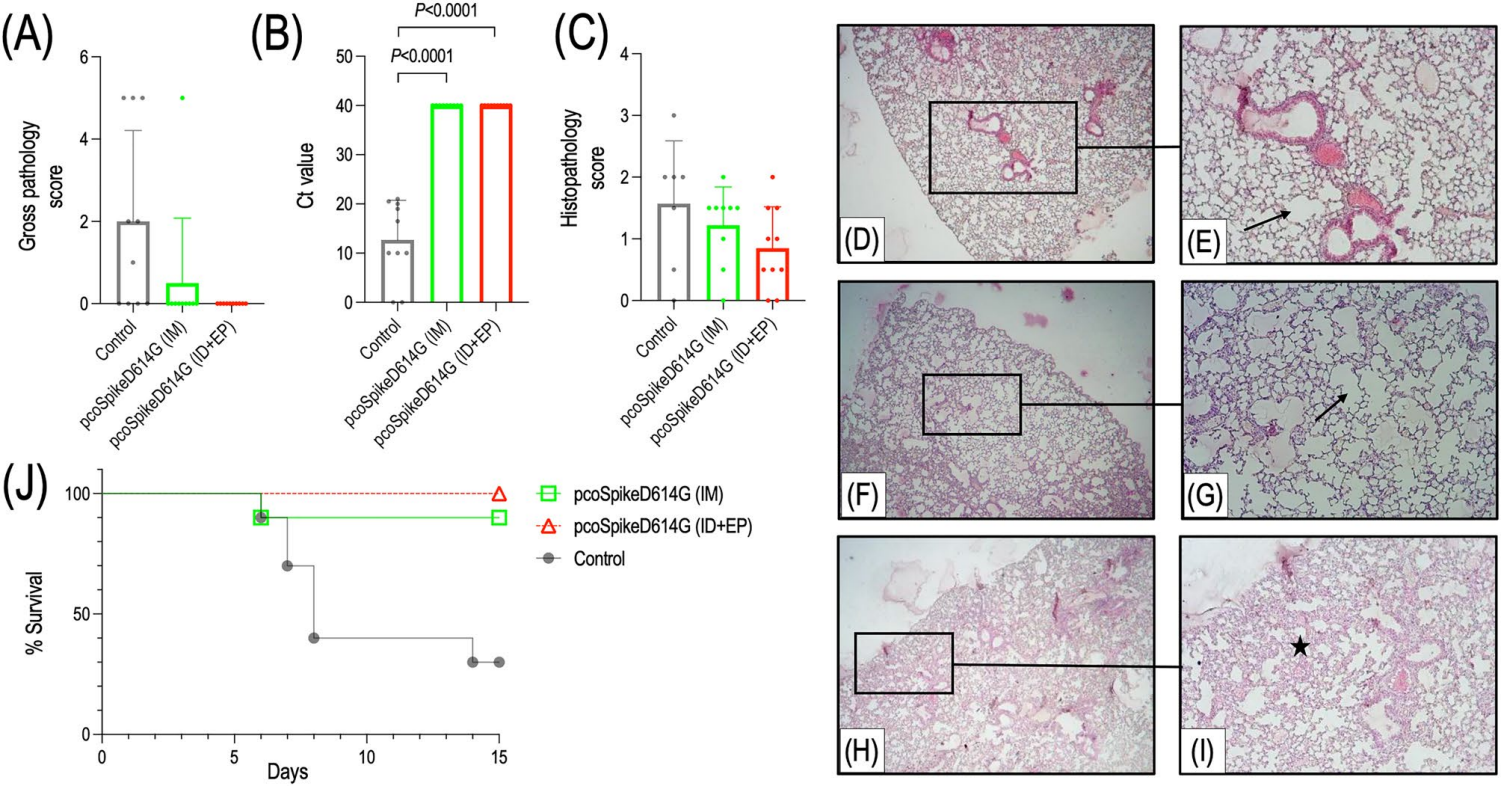
Protection conferred by pcoSpikeD614G DNA vaccine in K18-hACE2 transgenic mice challenged by SARS-CoV-2. (**A**) Gross pathological scoring of lungs to evaluate the level of pneumonia. Healthy lungs were scored as 0, edema-hyperemia between 0.5 and 1, pneumonia lesions were between 1.5 and 5 and dead mice were scored as 5. (**B**) Mean Ct values of RT-qPCR targeting NC gene of SARS‐CoV‐2 derived from the lungs of mice. The virus load is represented by Crossing point threshold (Ct) values. The Ct value of a negative PCR sample is accepted as 40 (**C**) Histopathology scoring of lungs to evaluate the level of inflammation status. Absence of inflammation is represented by 0 and various level of inflammation were scored between 1 and 3. Three lungs from the control group and one lung from the mice group immunized with pcoSpikeD614G administered through IM route were not scored (**D, E**) In the group of mice immunized with pcoSpikeD614G administered through IM route, images show normal lungs at magnifications of 4 × and 10 × . Arrow shows alveoli with normal morphology (**F, G**) In the group of mice immunized with pcoSpikeD614G administered through ID + EP route, images show normal lungs at magnifications of 4 × and 10 × . Arrow shows alveoli with normal morphology. (**H, I**) In the control group of mice, images show lungs with interstitial inflammatory cell infiltration at magnifications of 4 × and 10 × . Asterisks indicate areas of lymphocyte infiltration in the interalveolar spaces. (**J**) Kaplan–Meier Survival analysis after intranasal instillation of 10^5^ TCID_50_ virus for 3 consecutive days.

Two weeks after third vaccination, K18-hACE2 transgenic mice were challenged intranasally with 10^5^ TCID_50_ virus for 3 consecutive days. 15 days after instillation, lungs were extracted from the mice for gross pathological and histopathological examination, and in addition RT-qPCR targeting nucleocapsid (NC) gene of SARS‐CoV‐2 was performed to assess the virus load in lungs of mice. According to the gross pathology scoring, in the lungs of the mice immunized by pcoSpikeD614G administered through ID + EP route (n = 10) and by pcoSpikeD614G administered through IM route (n = 9), the organ integrity was intact, healthy, and pink in color. In the control group, there was moderate pneumonia in the lungs of mice (Fig. 5A). Regarding the mice that became ill and died before the end of the experiment, the pneumonia findings showing the course of the disease did not reach a level that could be seen with the naked eye. Visible pathological changes were observed in the lungs of some of the control group animals. Specifically, the lungs were edematous, hyperemic, and gray/brown color changes were observed representing the disruption of integrity at the microscopic level. The mean gross pathological scores were 0 and 0.5 for mice immunized by pcoSpikeD614G administered through ID + EP and IM routes compared to the control group with a mean score of 2 (Fig. 5A). During RT-qPCR, the virus load in the lungs of mice was represented by Ct (crossing point threshold) values. Accordingly, the virus load in lungs of control group mice was significantly higher than mice immunized by pcoSpikeD614G administered through IM and ID + EP (*P* < 0.0001) routes (Fig. 5B). Histopathology scoring of the lungs showed that the level of inflammation in the control group, pcoSpikeD614G administered through IM and ID + EP routes were 1.57, 1.2, and 0.85, respectively (Fig. 5C). Histopathological lung examination of the control group mice showed areas of lymphocyte infiltration in the interalveolar spaces (Fig. 5H–I) whereas mice immunized by pcoSpikeD614G administered through IM and ID + EP routes did not show any sign of inflammation in alveoli (Fig. 5D, E and F–G). After intranasal instillation of 10^5^ TCID_50_ virus for 3 consecutive days, 30% of control group, 90% of mice immunized with pcoSpikeD614G administered through IM route, and 100% of mice immunized with pcoSpikeD614G administered through ID + EP route survived (Fig. 5J).

### Preliminary study for large-scale DNA vaccine production

**Figure 6:**
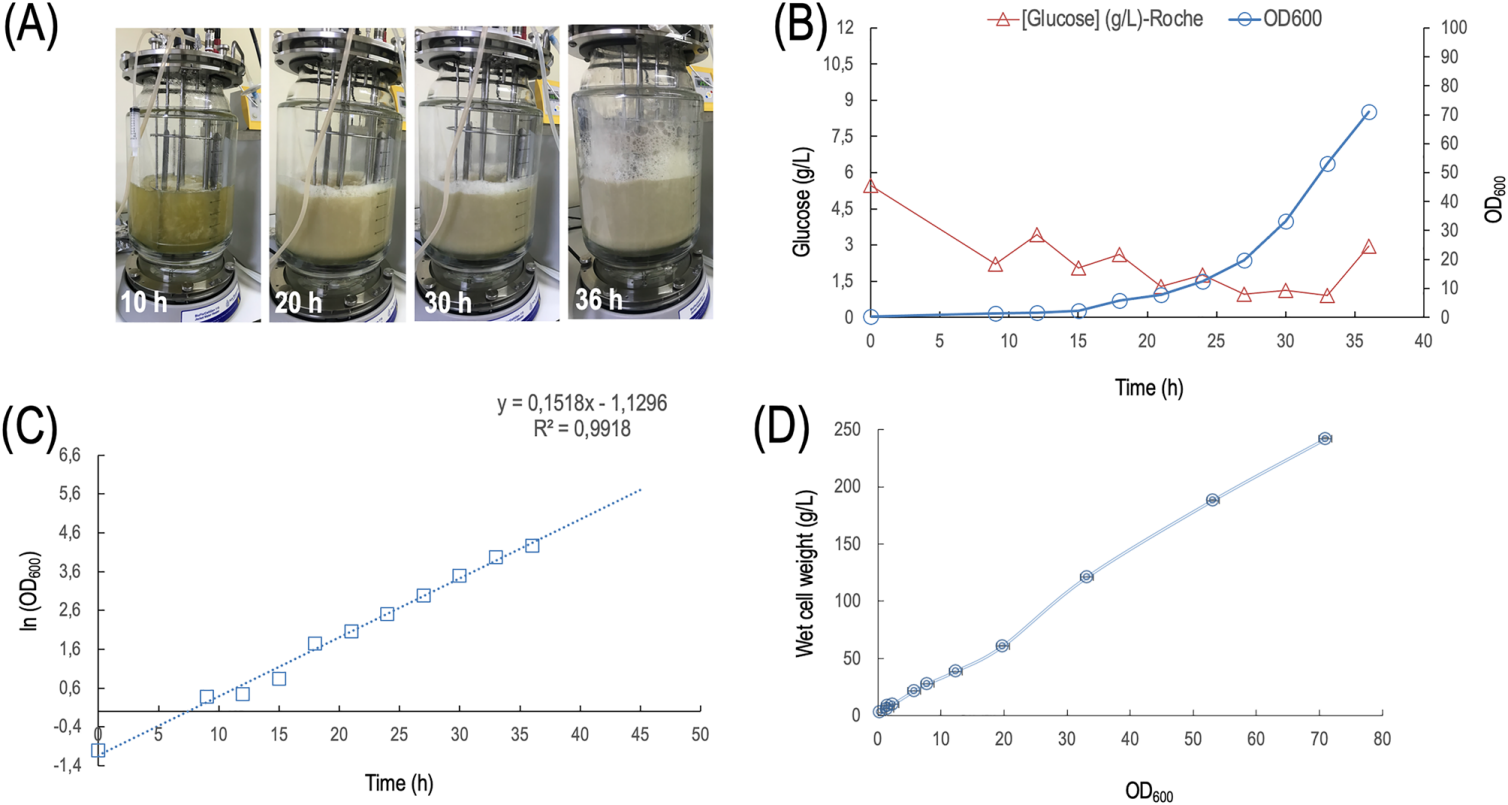
Large-scale production of *E. coli* DH5α containing DNA vaccine using chemically defined medium. (**A**) Representation of the medium inside 10 L bioreactor vessel becoming increasingly turbid at 10th hour, 20th hour, 30th hour and 36th hour of fermentation process (**B**) Graph showing the level of glucose concentration and OD_600_ over the course of 36 h of fermentation (**C**) Graph showing growth curve plotted on a log scale where a linear trend line was applied and the slope of which was obtained as the specific growth rate, μ (h^-1^) (**D**) Correlation between wet cell weight (g/L) and OD_600_ value.

After the detection of high immunogenicity and protection levels, large-scale upstream and downstream process optimization studies were conducted. For upstream process, 10 L bioreactor vessel and chemically defined medium were used to cultivate *E. coli* DH5α cells containing DNA vaccine. Samples were collected periodically to monitor cell growth by OD_600_ and glucose concentration. During the fermentation process, the medium in the 10 L vessel became increasingly turbid as shown in Fig. 6A. As shown in Fig. 6B, feeding was initiated at the 10th hour of fermentation when the glucose concentration dropped below 2 g/L and OD_600_ value reached 70.95 at the 36th hour of fermentation. The growth curve was plotted on a log scale to calculate the specific growth rate (μ = 0.15 h^-1^, Fig. 6C). Wet cell weight (WCW) was also calculated at various time points during the fermentation which increased proportionally with the increase in OD_600_ value. WCW reached 242 g/L at the 36th hour of fermentation (Fig. 6D).

**Figure 7:**
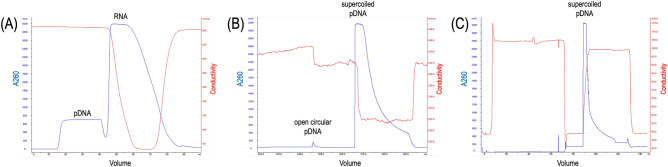
Purification of supercoiled DNA vaccine from clarified alkaline lysate using three-step chromatography. (**A**) RNA separation from DNA vaccine by size exclusion chromatography (**B**) Capturing supercoiled DNA vaccine and separation from open circular DNA vaccine using hydrophobic interaction column (**C**) Polishing of supercoiled DNA vaccine using anion exchanger column.

For downstream processing, alkaline lysis and three-step column chromatography were performed sequentially. After the alkaline lysis step, filtered and clarified supernatant was loaded to a size exclusion column to remove RNA by group separation, repeatedly until the supernatant is finished. During chromatography, the first fraction containing DNA vaccine was collected for the second step of purification, and subsequent large fraction containing RNA was discarded (Fig. 7A). In the second step of purification, the fraction containing DNA vaccine from the first step was loaded to a hydrophobic interaction column to separate open circular and supercoiled DNA vaccine. During chromatography, the first fraction containing open circular DNA was discarded and subsequent large fraction containing supercoiled DNA vaccine was collected for the third step of purification (Fig. 7B). In the third purification step, the fraction containing supercoiled DNA vaccine from the second step was loaded into an anion exchanger column to polish DNA vaccine (Fig. 7C). The final purified supercoiled DNA vaccine is concentrated with isopropanol and washed with ethanol using centrifugation and resuspended with 1 × PBS. Thereafter final purified supercoiled DNA vaccine was visualized with agarose gel electrophoresis which showed similar band pattern as shown in Fig. 2B (S6 Figure), and kept at − 20 °C.

## Discussion

COVID-19 is a global pandemic caused by SARS-CoV-2. After the identification of the novel virus in December 2019, in the Chinese city of Wuhan, it became a public emergency concern as declared by WHO in January, 2020 and nowadays it is accepted as endemic disease. Vaccines have played an important role in stopping the rapid spread of the virus all over the world such as mRNA vaccines including BNT162b2 (Pfizer/BioNTech) and mRNA-1273 (Moderna); non-replicating viral vector vaccines including AZD1222 (AstraZeneca), Ad26.COV2.S (Johnson & Johnson), and Covishield (Serum Institute of India); inactivated virus vaccines including BBIBP-CorV (Beijing Institute, Sinopharm), CoronaVac (Sinovac), Covaxin (Bharat Biotech, ICMR); protein subunit vaccines including Nuvaxovid (Novavax) and Covavax (Serum Institute of India) have been approved by the WHO.

Efficient DNA vaccines encoding Spike protein have been developed against SARS-CoV or MERS-CoV previously and they have entered clinical trials^38,39^, and during the COVID-19 pandemic, according to the WHO’s landscape of vaccine candidates, there were 33 DNA vaccine candidates in various phases of pre-clinical and clinical development^16^.

DNA vaccines have gained importance during the pandemic due to their advantages such as induction of strong immune response, rapid development, scalability, stability, and safety profiles. Notably, on August 20th, 2021, ZyCoV-D became the first approved DNA vaccine for humankind by the Drug Controller General of India^9,40^.

In this study, we developed a DNA vaccine encoding Spike protein administered through IM and ID routes using an electroporator, also listed in the WHO’s landscape of DNA vaccine candidates against COVID-19 (developer is Ege University). To design the Spike antigen, we analyzed the presence of mutations introduced to the *Spike* gene of SARS-CoV-2 in clinical samples collected from COVID-19 patients living in 8 different provinces of Türkiye. Among the mutations analyzed, A1841G mutation leading to D614G alteration in amino acid sequence was detected in 85% of the isolates (Fig. 1A,B). This non-synonymous D614G was detected in all the VOC (Alpha, Beta, Gamma, and Delta) during the pandemic as well as the current VOC Omicron in circulation^41^. D614G mutation made Spike become more open while increasing its binding affinity and interaction with the ACE2 receptor. This high prevalence of D614G has also conferred a selective advantage for the SARS-CoV-2 and made its detection widespread^42^. Specifically, in a study it has been shown that the D614G mutation increases virus entry into the host cell while maintaining its neutralization susceptibility, resulting in high transmission and low mortality, ensuring the persistence of SARS-CoV-2 in humanity^43^. So, we included this mutation into our antigen design and docked it with the human ACE2 receptor. The docking results also showed stronger binding affinity between coSpikeD614G protein and the human ACE2 receptor than the Wuhan Spike protein (Fig. 1C). Thereafter, we constructed the DNA vaccine by fusing coSpikeD614G protein in frame with IGHE signal peptide using pVAX1 as a vector backbone. The protein expression capacity of pcoSpikeD614G DNA vaccine was tested by in vitro transfection of HEK293T cells and results of IFAT, Western blot, and RT-qPCR assays showed that coSpikeD614G protein was abundantly expressed (Fig. 2C–J). Then, immunogenicity and protection studies were conducted in BALB/c and transgenic K18-hACE2 mice immunized thrice with pcoSpikeD614G administered through IM route and ID route using an electroporator device (Fig. 3A). During the analyses of the humoral immune response elicited by pcoSpikeD614G in sera of BALB/c mice, Western blot showed the presence of anti-S1 and anti S1 + S2 specific IgG antibody responses, ELISA showed significantly higher anti-S1 IgG responses polarized through IgG2a indicating Th1 biased immune response (Fig. 3B–D). Surrogate Virus Neutralization Test and microneutralization assay showed high inhibition potential of neutralizing antibodies compared to controls (*P* < 0.0001) (Fig. 3F,G). During the analyses of the cellular immune response elicited by pcoSpikeD614G in splenocyte culture of BALB/c mice, extracellular IFN-γ levels induced by pcoSpikeD614G DNA vaccine have significantly increased in mice administered through IM (*P* = 0.0029) or ID + EP (*P* < 0.0001) routes (Fig. 4A). Flow cytometry analyses showed that in the mice group immunized with pcoSpikeD614G administered through IM and ID + EP routes, the mean ratio of CD8^+^ cells secreting IFN-γ increased by %5.24 and %12.51, and the mean ratio of CD4^+^ cells secreting IFN-γ increased by %3.78 and %10.19 (Fig. 4B, C). The main essential facet of immune response against SARS-CoV-2 includes B cells differentiating into plasma cells which secretes neutralizing antibodies (such as IgA, IgM and IgG), CD4^+^ T helper cells, and CD8^+^ killer T cells^44^. Similar to the host immune response after vaccinating mice through IM route or ID route using electroporation, the pcoSpikeD614G DNA vaccine induced strong humoral immune response as shown by significant increase in anti-S1 IgG and neutralizing antibodies as well as cellular response as shown by significant increase in CD4^+^ and CD8^+^ cells secreting IFN-γ in BALB/c mice. The challenging studies conducted in transgenic K18-hACE2 mice immunized with pcoSpikeD614G administered through IM and ID + EP routes showed that after intranasal instillation of 10^5^ TCID_50_ virus for 3 consecutive days, 90% and 100% of mice survived (Fig. 5J). Importantly, RT-qPCR did not detect any virus load in the lungs of survived mice immunized by pcoSpikeD614G administered through ID + EP and IM routes (Fig. 5B). During gross pathology and histopathological examination, the lung integrity was healthy and did not show signs of inflammation in mice immunized by pcoSpikeD614G administered through ID + EP or IM routes. To summarize, pcoSpikeD614G DNA vaccine administered through ID route using an electroporator has shown slightly higher immunogenicity and protection levels compared to those administered through IM route. Specifically, there was not a significant difference between the level of neutralizing antibodies and the increase in CD8^+^ cells secreting IFN-γ achieved by pcoSpikeD614G DNA vaccine administered through ID + EP route was significantly higher than the IM route (*P* = 0.0023). The protective efficacies of both vaccines were comparable except for a mouse that died on day 6 of challenging in the group of mice immunized with pcoSpikeD614G DNA vaccine administered through IM route.

Numerous DNA vaccine studies have been conducted against SARS-CoV-2 mainly using codon optimized *Spike* gene as an antigen. Several plasmid backbones were used during the DNA vaccine construction including pVAX, pcDNA3.1, pCMVkan, pGX27, NTC8685-eRNA41H or pTK1A-TPA. DNA vaccines have been either administered alone or by the help of electroporation devices or needle free injection systems to increase the immunogenicity and protection levels in preclinical studies (Table 1). The WHO's list of DNA vaccine candidates against COVID-19 includes 33 DNA vaccine candidates that differ from each other in terms of antigen design, plasmid used, delivery method, route of administration or amount of DNA applied. Some of the preclinical studies on this list have moved on to clinical trials, such as INO-4800^56^ or ZyCoV-D^57^, due to their protective efficacy in animal studies. In addition to success in animal studies, those entering clinical trials have both an already established DNA vaccine platform, an effective delivery device and more experience in clinical trials. The results of these studies and our study showed that DNA vaccines encoding full *Spike* gene codon optimized for expression in mammalian cells are inducing protective humoral response and Th1 biased cellular immune response and conferred high level of protection against SARS-CoV-2 in various animal models including non-human primates. Moreover, direct administration of DNA vaccine using IM route or administration of DNA vaccine followed by electroporation are the most frequently preferred immunization routes in pre-clinical studies. The use of electroporation in clinical trials of DNA vaccines has some disadvantages, including low patient acceptance due to the invasiveness and discomfort of the procedure, safety concerns, adverse reactions, limited scalability for mass vaccination programs, the need for high-tech equipment for implementation, and high cost and regulatory challenges for integration into health systems^58^.

**Table 1:** DNA vaccine studies against COVID-19.

Antigen	Strain	Plasmid backbone	Vaccination route	Animal model	Efficiency	References
Full spike	Wuhan	pVAX	IM + EP (Cellectra)	Mouse and pig	INO-4800 vaccine conferred high level of protective humoral and cellular immune responses	^30^
Full spike	Wuhan	pVAX	ID + EP (Cellectra)	Rhesus macaque	INO-4800 vaccine elicited T-cells secreting IFN-γ, conferred lower viral loads in the monkey lungs	^45^
Full spike	Wuhan	pcDNA3.1	IM	Rhesus macaque	DNA vaccine Induced humoral and cellular immune responses including high levels of neutralizing Ab, and protection against SARS-CoV-2	^46^
Full spike	Wuhan	pcDNA3.1	Gene Gun (Helios) + Recombinant S1 protein	Rhesus monkey	DNA vaccine elicited high level neutralizing antibodies and T cell immune response	^47^
Full spike	–	pCMVkan	IM + EP (TriGrid)	Mouse	DNA vaccine induced high level of IgG Ab, balanced IgG1/IgG2a response, neutralizing Ab, and T cells secreting IFN-γ	^48^
Full spike	Wuhan	pGX27	IM + EP (OrbiJector)	Mouse and Cynomolgus macaque	GX-18 DNA vaccine induced 1) high level of Anti-Spike IgG Ab and Th1-biased T cell responses in mice; 2) neutralizing Ab, multifunctional CD4^+^ and CD8^+^ T cell responses and reduced viral loads in Cynomolgus macaques	^49^
Full spike	Wuhan	pVAX1	IM	Syrian hamster	DNA vaccine induced SARS-CoV-2-specific neutralizing Ab titers and decreased lung viral loads, but lung pathology was similar to controls	^50^
Full spike	Wuhan	pVAX	IM + EP (BTX)	Mouse and hamster	DNA vaccine induced anti-RBD Ab, very high titers of neutralizing Ab, Th1 biased cellular response and conferred protection in hamsters	^51^
Full spike	Wuhan	pVAX1	ID	Mouse and guinea pig	ZyCoV-D vaccine induced high IgG Ab and neutralizing Ab responses in all animals and conferred elevated IFN-γ response in mice splenocyte culture	^52^
			ID + PharmaJet Tropis needle free injection	New Zealand white rabbit		
Full spike	Wuhan	pVAX1	ID + PharmaJet Tropis needle free injection	Rhesus macaques	ZyCoV-D vaccine elicited significantly high titers of specific IgG and neutralizing Ab, increased cellular immune response and conferred protection	^53^
Full spike	Wuhan	NTC8685-eRNA41H	ID	Mouse	DNA vaccine induced Spike-specific IgG and neutralizing antibodies in mice, rabbits, and Rhesus macaques together with robust Th1 dominant cellular responses, and a challenge study of Rhesus macaques demonstrated protection from viral replication in the lungs	^54^
			ID + PharmaJet Tropis needle free injection	Rabbit		
			IM + PharmaJet Stratis needle free Injection	Rabbit		
			ID + PharmaJet Tropis needle free Injection	Rhesus macaques		
RBD	Wuhan	pTK1A-TPA	IM + EP (IGEA Cliniporator)	Mouse, rat, and ferret	COVID-eVax vaccine induced potent neutralizing Ab and robust T cell response, and conferred protection in K18-hACE2 transgenic mice and ferrets	^55^

Although EP is preferred in some DNA vaccine studies against COVID-19 (Table 1), it takes a long time to optimize the EP device, especially the voltage regime, the amount of DNA to be administered or the route of administration. On the other hand, IM administration of DNA vaccines can also provide good immunogenicity data without much optimization. And in this study, although administration of pcoSpikeD614G DNA vaccine administered through ID + EP induced slightly higher humoral and CD8^+^ T cell response compared to IM route, protective efficacies of both vaccines were comparable which shows that administration of the pcoSpikeD614G DNA vaccine through IM route can also be preferred in clinical studies.

In this study, after detection of high immunogenicity and protection levels, we further developed a large-scale production process. For this purpose, we used a modified chemically defined medium and a bioreactor for upstream large-scale production of DNA vaccine^36^. During late clinical trials, 100 mg of DNA vaccine is required due to usage of approximately 1 mg/dose. Fermentation in Erlenmeyer flask with typical yields of 10–20 mg/L becomes impractical^59^. For this reason, fed-batch fermentation method especially useful for plasmid production was used in this study^59^. After the optimization studies, we defined a 36-h fermentation process in which WCW reached 242 g/L. DeLisa et al. (1998) used fed-batch fermentation to produce *E. coli* JM105 strain in which DCW (dry cell weight) reached 110 g/L using the chemically defined medium which was also used in this study^36^. In an application note, WCW reached maximum 466 g/L (DCW: 113.1 g/L)^60^. For this reason, more optimization studies are required to increase the yield of our upstream processing to be more cost effective. For downstream processing, we used an alkaline lysis method preceded by a three-step column chromatograph adopted from an application note in which 4560 μg/ml of pDNA is obtained after last filtration step (98% is supercoiled plasmid)^37^. Similarly, in this study, filtered and clarified supernatant from alkaline lysis was loaded to a size exclusion column to remove RNA, and then hydrophobic interaction column was used to capture supercoiled DNA vaccine, and lastly an anion exchanger column was used to polish and obtain purified DNA vaccine. The agarose gel image of the purified pDNA product contains a faint band on top of supercoiled pDNA band (S6 Figure). According to the guidance document named “Considerations for Plasmid DNA Vaccines for Infectious Disease Indications” published by FDA in 2007, a minimum specification for supercoiled plasmid content according to batch release criteria should preferably be > 80%^61^ and in future studies further analyses can be performed such as HPLC to determine the percentage of purified supercoiled pDNA. As a result of this preliminary study for large-scale DNA vaccine production, progress towards the next step in the DNA vaccine development pipeline was achieved.

## Conclusion

Vaccines against SARS-CoV-2 would possess some key properties such as they should have high efficacy, long lasting immunity by inducing both arms of immune response, broad protection against all VOCs, ease of adaptation to new variants by quick development, ease of administration, stability under standard storage conditions, and be easily scalable and safe. As it can be assessed from the previous studies and the results of this study, DNA vaccines are promising vaccine development platforms that can address these basic needs related to SARS-CoV-2 and can be a convenient option in future pandemics. At this point, it is noteworthy that pcoSpikeD614G DNA vaccine induced high levels of neutralizing antibody, strong Th1 biased immune response, and conferred high protection in preclinical studies against SARS-CoV-2 and moreover, a large-scale production process of the pcoSpikeD614G DNA vaccine was described in this study. In addition, this vaccine platform can be used in other non-communicable diseases such as cancer in which a Th1 biased cellular immune response is critical. Finally, although the pcoSpikeD614G DNA vaccine developed in this study provided a high protective immune response and protection against the D614G variant, its protection efficacy against current VOCs is questionable and future studies may be conducted to investigate the efficacy of this vaccine against new variants of SARS-CoV-2.

### Supplementary Information

Supplementary Information.

## Data Availability

The datasets used and analyzed during the current study are available from the corresponding author upon reasonable request.
