# Supplementary material for: Immunogenicity and protection efficacy of a COVID-19 DNA vaccine encoding spike protein with D614G mutation and optimization of large-scale DNA vaccine production
Source: Sci Rep. 2024 Jun 15;14:13865. doi: 10.1038/s41598-024-64690-5 (PMC11180131; doi:10.1038/s41598-024-64690-5)

**Supplementary file**

**S1 Table.** Sequencing primers for Spike gene of SARS-CoV-2 designed by primer designing tool (NCBI; <https://www.ncbi.nlm.nih.gov/tools/primer-blast/>)

| **Primer pairs** | **Product size (bp)** |
| --- | --- |
| F1: 5-AAGGGGTACTGCTGTTATGTCTT-3  R1: 5-CAAGGTCCATAAGAAAAGGCTGA-3 | 681 |
| F2: 5-TTGTAATGATCCATTTTTGGGTGT-3  R2: 5-TTCTCTTCCTGTTCCAAGCAT-3 | 668 |
| F3: 5-TTGTGCCCTTTTGGTGAAGT-3  R3: 5-AAGAACAGCAACCTGGTTAGA-3 | 831 |
| F4: 5-CGTGATCCACAGACACTTGAGA-3  R4: 5-TGTCTTGGTCATAGACACTGGT-3 | 474 |
| F5: 5-GGCTGAACATGTCAACAACTCA-3  R5: 5-CACCAAAGGTCCAACCAGAAG-3 | 713 |
| F6: 5-ACACTTCTGCACTGTTAGCG-3  R6: 5-GCCCTTTCCACAAAAATCAACT-3 | 521 |
| F7: 5-AGAGTGTGTACTTGGACAATCA-3  R7: 5-GCATCCTTGATTTCACCTTGC-3 | 809 |

**S2 Table.** Recombinant coSpike614G protein expressed by pcoSpikeD614G (1290 aa) with a theoretical molecular weight of 143.088 kDa

| **Recombinant coSpikeD614G protein expressed by pcoSpikeD614G (1290 aa, 143.1 kDa)** |
| --- |
| Underlined region: the signal peptide of Homo sapiens Ig heavy chain epsilon-1 (V-D-J region) (IGHE)  Total size of the ORF is 1290 aa  MDWTWILFLVAAATRVHSFVFLVLLPLVSSQCVNLTTRTQLPPAYTNSFTRGVYYPDKVFRSSVLHSTQDLFLPFFSNVTWFHAIHVSGTNGTKRFDNPVLPFNDGVYFASTEKSNIIRGWIFGTTLDSKTQSLLIVNNATNVVIKVCEFQFCNDPFLGVYYHKNNKSWMESEFRVYSSANNCTFEYVSQPFLMDLEGKQGNFKNLREFVFKNIDGYFKIYSKHTPINLVRDLPQGFSALEPLVDLPIGINITRFQTLLALHRSYLTPGDSSSGWTAGAAAYYVGYLQPRTFLLKYNENGTITDAVDCALDPLSETKCTLKSFTVEKGIYQTSNFRVQPTESIVRFPNITNLCPFGEVFNATRFASVYAWNRKRISNCVADYSVLYNSASFSTFKCYGVSPTKLNDLCFTNVYADSFVIRGDEVRQIAPGQTGKIADYNYKLPDDFTGCVIAWNSNNLDSKVGGNYNYLYRLFRKSNLKPFERDISTEIYQAGSTPCNGVEGFNCYFPLQSYGFQPTNGVGYQPYRVVVLSFELLHAPATVCGPKKSTNLVKNKCVNFNFNGLTGTGVLTESNKKFLPFQQFGRDIADTTDAVRDPQTLEILDITPCSFGGVSVITPGTNTSNQVAVLYQGVNCTEVPVAIHADQLTPTWRVYSTGSNVFQTRAGCLIGAEHVNNSYECDIPIGAGICASYQTQTNSPRRARSVASQSIIAYTMSLGAENSVAYSNNSIAIPTNFTISVTTEILPVSMTKTSVDCTMYICGDSTECSNLLLQYGSFCTQLNRALTGIAVEQDKNTQEVFAQVKQIYKTPPIKDFGGFNFSQILPDPSKPSKRSFIEDLLFNKVTLADAGFIKQYGDCLGDIAARDLICAQKFNGLTVLPPLLTDEMIAQYTSALLAGTITSGWTFGAGAALQIPFAMQMAYRFNGIGVTQNVLYENQKLIANQFNSAIGKIQDSLSSTASALGKLQDVVNQNAQALNTLVKQLSSNFGAISSVLNDILSRLDKVEAEVQIDRLITGRLQSLQTYVTQQLIRAAEIRASANLAATKMSECVLGQSKRVDFCGKGYHLMSFPQSAPHGVVFLHVTYVPAQEKNFTTAPAICHDGKAHFPREGVFVSNGTHWFVTQRNFYEPQIITTDNTFVSGNCDVVIGIVNNTVYDPLQPELDSFKEELDKYFKNHTSPDVDLGDISGINASVVNIQKEIDRLNEVAKNLNESLIDLQELGKYEQYIKWPWYIWLGFIAGLIAIVMVTIMLCCMTSCCSCLKGCCSCGSCCKFDEDDSEPVLKGVKLHYT- |

**S3 Table**. Immunization Groups, Doses, and Schedule

| **Groups** | **Mice Model** | **Numbers of Mice** | **Route of Administration** | **Doses**  **(µg plasmid/dose)** | **Schedule** | **Study** |
| --- | --- | --- | --- | --- | --- | --- |
| pcoSpikeD614G | BALB/c | 15 | IM | 100 | Day 0+14+56 | Immunogenicity |
| Empty PVAX1 (C) | BALB/c | 15 | IM | 100 | Day 0+14+56 |  |
| pcoSpikeD614G | BALB/c | 15 | ID+EP* | 25 | Day 0+14+56 |  |
| Empty PVAX1 (C) | BALB/c | 15 | ID+EP* | 25 | Day 0+14+56 |  |
| pcoSpikeD614G | K18-hACE2 transgenic | 10 | IM | 100 | Day 0+14+56 | Protective efficacy |
| pcoSpikeD614G | K18-ACE2 transgenic | 10 | ID+EP* | 25 | Day 0+14+56 |  |
| Empty PVAX1 (C) | K18-hACE2 transgenic | 10 | IM | 100 | Day 0+14+56 |  |
| *Electroporation was performed with a 4 mm gap electrode using the following pulse protocol: first voltage group 450V, 0.05 ms pulse length, 0.2 ms pulse interval using one pulse; second voltage group 450V, 0.05 ms pulse length, 50 ms pulse interval using one pulse; third voltage group 110V, 10 ms pulse length, 10 ms pulse interval using eight pulses. | | | | | | |

**S4 Table**. Spike Protein Immunogenic Peptides Used to Stimulate Splenocyte Culture (Smith et. al, 2020; Can et al., 2020). The peptides are underlined inside the ORF of recombinant coSpikeD614G protein expressed by pcoSpikeD614G.

VVLSFELLHAPATVC

PHGVVFLHVTYVPAQ

DDFTGCVIAWNSNNL

FTTAPAICHDGKAHF

KNKCVNFNFNGLTGT

VGGNYNYLYRLFRKS

KLNDLCFTNV

| **Recombinant coSpikeD614G protein expressed by pcoSpikeD614G (1290 aa, 143.1 kDa)** |
| --- |
| Total size of the ORF is 1290 aa  MDWTWILFLVAAATRVHSFVFLVLLPLVSSQCVNLTTRTQLPPAYTNSFTRGVYYPDKVFRSSVLHSTQDLFLPFFSNVTWFHAIHVSGTNGTKRFDNPVLPFNDGVYFASTEKSNIIRGWIFGTTLDSKTQSLLIVNNATNVVIKVCEFQFCNDPFLGVYYHKNNKSWMESEFRVYSSANNCTFEYVSQPFLMDLEGKQGNFKNLREFVFKNIDGYFKIYSKHTPINLVRDLPQGFSALEPLVDLPIGINITRFQTLLALHRSYLTPGDSSSGWTAGAAAYYVGYLQPRTFLLKYNENGTITDAVDCALDPLSETKCTLKSFTVEKGIYQTSNFRVQPTESIVRFPNITNLCPFGEVFNATRFASVYAWNRKRISNCVADYSVLYNSASFSTFKCYGVSPTKLNDLCFTNVYADSFVIRGDEVRQIAPGQTGKIADYNYKLPDDFTGCVIAWNSNNLDSKVGGNYNYLYRLFRKSNLKPFERDISTEIYQAGSTPCNGVEGFNCYFPLQSYGFQPTNGVGYQPYRVVVLSFELLHAPATVCGPKKSTNLVKNKCVNFNFNGLTGTGVLTESNKKFLPFQQFGRDIADTTDAVRDPQTLEILDITPCSFGGVSVITPGTNTSNQVAVLYQGVNCTEVPVAIHADQLTPTWRVYSTGSNVFQTRAGCLIGAEHVNNSYECDIPIGAGICASYQTQTNSPRRARSVASQSIIAYTMSLGAENSVAYSNNSIAIPTNFTISVTTEILPVSMTKTSVDCTMYICGDSTECSNLLLQYGSFCTQLNRALTGIAVEQDKNTQEVFAQVKQIYKTPPIKDFGGFNFSQILPDPSKPSKRSFIEDLLFNKVTLADAGFIKQYGDCLGDIAARDLICAQKFNGLTVLPPLLTDEMIAQYTSALLAGTITSGWTFGAGAALQIPFAMQMAYRFNGIGVTQNVLYENQKLIANQFNSAIGKIQDSLSSTASALGKLQDVVNQNAQALNTLVKQLSSNFGAISSVLNDILSRLDKVEAEVQIDRLITGRLQSLQTYVTQQLIRAAEIRASANLAATKMSECVLGQSKRVDFCGKGYHLMSFPQSAPHGVVFLHVTYVPAQEKNFTTAPAICHDGKAHFPREGVFVSNGTHWFVTQRNFYEPQIITTDNTFVSGNCDVVIGIVNNTVYDPLQPELDSFKEELDKYFKNHTSPDVDLGDISGINASVVNIQKEIDRLNEVAKNLNESLIDLQELGKYEQYIKWPWYIWLGFIAGLIAIVMVTIMLCCMTSCCSCLKGCCSCGSCCKFDEDDSEPVLKGVKLHYT- |

| 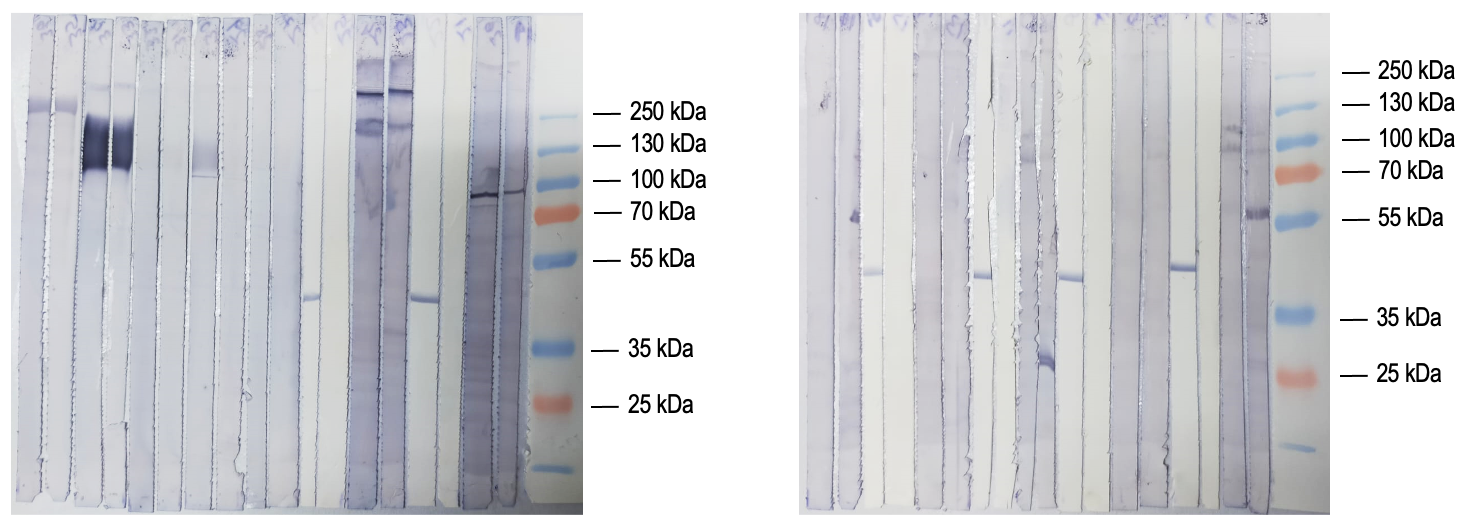 |  |
| --- | --- |

**S5 Figure.** Original Western blotting images used to generate Figure 2I and Figure 3B. The blots were cut prior to hybridization.

**S6 Figure.** Agarose gel image of final purified pDNA from the large-scale bioprocess


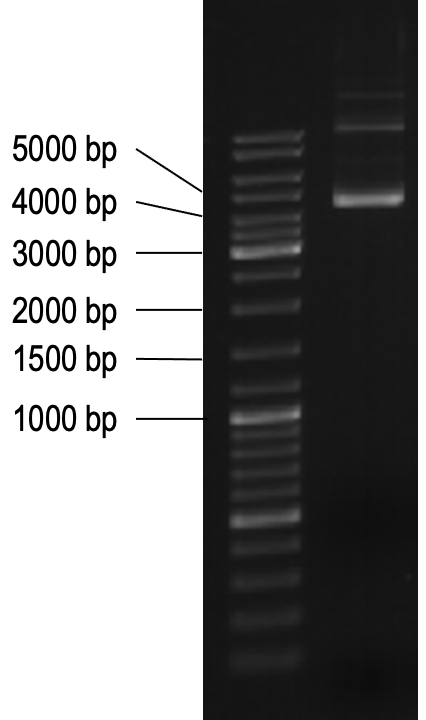

Supplement: Supplementary file 1 — Supplementary Information. [file 41598_2024_64690_MOESM1_ESM.docx]
